# Comparing Within- and Between-Family Polygenic Score Prediction

**DOI:** 10.1016/j.ajhg.2019.06.006

**Published:** 2019-07-11

**Authors:** Saskia Selzam, Stuart J. Ritchie, Jean-Baptiste Pingault, Chandra A. Reynolds, Paul F. O’Reilly, Robert Plomin

**Affiliations:** 1 Social, Genetic and Developmental Psychiatry Centre, Institute of Psychiatry, Psychology and Neuroscience, King’s College London, London SE5 8AF, UK; 2 Division of Psychology and Language Sciences, University College London, London WC1H 0AP, UK; 3 Department of Psychology, University of California Riverside, Riverside, CA 92521, USA; 4 Icahn School of Medicine, Mount Sinai, New York, NY 10029, USA

**Keywords:** polygenic score prediction, complex trait prediction, gene-environment correlation, genetic nurture, gene-environment interplay, within-family analysis, socio-economic status

## Abstract

Polygenic scores are a popular tool for prediction of complex traits. However, prediction estimates in samples of unrelated participants can include effects of population stratification, assortative mating, and environmentally mediated parental genetic effects, a form of genotype-environment correlation (rGE). Comparing genome-wide polygenic score (GPS) predictions in unrelated individuals with predictions between siblings in a within-family design is a powerful approach to identify these different sources of prediction. Here, we compared within- to between-family GPS predictions of eight outcomes (anthropometric, cognitive, personality, and health) for eight corresponding GPSs. The outcomes were assessed in up to 2,366 dizygotic (DZ) twin pairs from the Twins Early Development Study from age 12 to age 21. To account for family clustering, we used mixed-effects modeling, simultaneously estimating within- and between-family effects for target- and cross-trait GPS prediction of the outcomes. There were three main findings: (1) DZ twin GPS differences predicted DZ differences in height, BMI, intelligence, educational achievement, and ADHD symptoms; (2) target and cross-trait analyses indicated that GPS prediction estimates for cognitive traits (intelligence and educational achievement) were on average 60% greater between families than within families, but this was not the case for non-cognitive traits; and (3) much of this within- and between-family difference for cognitive traits disappeared after controlling for family socio-economic status (SES), suggesting that SES is a major source of between-family prediction through rGE mechanisms. These results provide insights into the patterns by which rGE contributes to GPS prediction, while ruling out confounding due to population stratification and assortative mating.

## Introduction

The recent influx of well-powered genome-wide association (GWA) studies has led to substantial advances in our ability to detect genetic associations between single base pair variants (single-nucleotide polymorphisms [SNPs]) across the genome and a myriad of complex traits. Although individual SNP effect sizes are extremely small,^1^ the surge in GWA power has improved the ability to predict complex traits through the genome-wide polygenic score (GPS) approach.^2^, ^3^ GPSs are indices of individuals’ genetic propensity for a trait and are derived as the sum of the total number of trait-associated alleles across the genome, weighted by their respective association effect size estimated through GWA analysis.^4^ GPS can be calculated in any sample with genotype data that is independent from the discovery GWA study, and have permeated research in the social, behavioral, and biomedical sciences.^5^ In this paper, we use within-family analysis to investigate an important potential source of prediction in polygenic score analysis: passive genotype-environment correlation.

Currently one of the largest GWA meta-analyses with a sample size of 1.1 million was performed on educational attainment (years of schooling).^6^ A GPS derived from this study is the most predictive GPS for any behavioral trait to date, explaining 10.6% of the variance in years of education^6^ and 14.8% in tested educational achievement.^7^ The predictive power of the educational attainment GPS (EA GPS) is considerable in contrast to other GPS for behavioral traits. Notably, cross-trait analyses have revealed that EA GPS is widely associated with traits other than educational achievement, including intelligence,^2^, ^6^, ^7^ socioeconomic status (SES),^8^, ^9^, ^10^, ^11^ behavior problems,^12^ mental health,^13^ physical health,^13^ and personality,^14^, ^15^ in some cases accounting for as much as or more than the variance in cross-trait associations explained by the target GPSs themselves.^15^, ^16^

However, GWA analyses, and the GPSs derived from them in independent samples, are naive to the pathways that lead from SNPs to trait outcomes.^17^ With a focus on prediction, the mechanisms by which polygenic scores relate to phenotypes are left largely unexplored. Given the popularity and widespread use of the GPS approach, the interpretation of GPS prediction estimates requires more careful consideration. Potentially, *passive genotype-environment correlation* (prGE)^18^ effects could be one source of prediction. Parents generate family environments consistent with their own genotypes, which in turn facilitate the development of the offspring trait, thus inducing a correlation between offspring genotype and family environment.^19^, ^20^, ^21^ Although these effects are also genetic in origin, they stem from the parents and are thus environmentally mediated. Therefore, GPS prediction among unrelated individuals may include contributions from both direct genetic effects and also indirect effects due to prGE.

Within-family analysis of siblings is a powerful approach to disentangle these potential sources of prediction. The additive genetic correlation between siblings is on average 0.50,^22^ and the transmission of alleles from parents to offspring is randomized during meiosis, such that siblings have equal probability of inheriting any given allele.^23^ The variability around the average genetic relationship between siblings due to random segregation is generally independent of the environment, so any genetic difference between siblings is free of shared environmental influence.^24^ A relationship between their genetic differences and trait differences provides evidence for a causal effect of the measured genetic difference, since (1) siblings are well matched on all shared familial genetic influences that shape the environment, and (2) potential bias due to population stratification and assortative mating is completely eliminated within families.^6^, ^25^, ^26^ Such within-family analyses account for prGE effects that are related to common family environments that are correlated with the transmitted alleles shared between siblings, but also environmental effects related to non-transmitted parental alleles that contribute to offspring similarity within a family. The use of DZ co-twins strengthens this design further as all shared environmental influences are time-invariant between twins (e.g., pregnancy risk factors, parental age, family income).

Indeed, previous within-family analyses have revealed substantial reductions in individual SNP effect sizes. For example, there was an effect size attenuation of ∼40% compared to between-family associations in the most recent GWA study on educational attainment.^6^ Most of this reduction has been attributed to prGE; no similar deflation of effect sizes was found for height,^6^ indicating that prGE is not likely at play. A novel method relying on closely and distantly related individuals, and that is applied to very large populations, detected a similar reduction of SNP-heritability estimates of educational achievement (∼40%).^24^ Moreover, studies that tested the effect between non-transmitted alleles from parental to offspring genotypes on offspring outcomes reported a significant association for educational attainment^20^, ^21^—an effect of so-called *genetic nurture*—but not for height and BMI.^20^, ^21^ In contrast, one study that tested within-family predictions of educational attainment using the EA GPS found no noteworthy difference in comparison to between-family estimates.^27^ However, this GPS was based on the first GWA study for educational attainment^28^ and may have been underpowered to pick up prGE-driven effects. Indeed, a more recent study found that using the latest GPS for educational attainment, there was an attenuation of ∼55% in the prediction of years of schooling within families in comparison to between-family estimates.^29^

Overall, relatively little research has been conducted on within-family GPS prediction, mostly focusing on educational and anthropometric traits. This study adds substantially to this literature by systematically comparing within-family GPS prediction to between-family GPS prediction across eight life outcomes (height, BMI, self-rated health, intelligence, educational achievement, neuroticism, attention-deficit/hyperactivity symptoms, and schizophrenia symptoms). Educational achievement is both phenotypically and genetically correlated with many life outcomes.^30^, ^31^, ^32^, ^33^, ^34^, ^35^, ^36^ It is also highly genetically correlated with family SES,^8^, ^37^, ^38^ and EA GPS predicts 7.3% of the variance in SES.^9^ Therefore, it is possible that the effects identified in the GWA studies for educational attainment related to family environment (e.g., SES) also contribute to the development of other behavioral traits through prGE mechanisms. Although it has been suggested that the widespread cross-trait associations between the EA GPS and various outcomes may be partly driven by prGE effects,^15^, ^39^ to our knowledge no study to date has tested this hypothesis.

It is the aim of this study to investigate potential influences of prGE in a range of life outcomes through the comparison of within- and between-family polygenic score prediction estimates. First, we predict that within-family estimates will be disproportionally lower than between-family estimates for EA GPS predictions of educational achievement in contrast to other GPS predictions of their target trait. Second, we predict that cross-trait associations between the EA GPS and other outcomes will be smaller within families than between families, in comparison to the cross-trait associations of other GPSs.

## Material and Methods

Our hypotheses, measures, and analysis plan were preregistered with the Open Science Framework (for more details, see Web Resources), except where indicated below. The non-preregistered analyses should be considered exploratory.

### Sample

Participants were drawn from the Twins Early Development Study (TEDS). Between 1994 and 1996, TEDS recruited 16,810 twin pairs born in England and Wales, who have been assessed in multiple waves across development until the present. The demographic characteristics of TEDS participants and their families closely match those of families in the UK.^9^, ^40^ Written informed consent was obtained from parents prior to data collection and from TEDS participants themselves past the age of 18. Project approval was granted by King’s College London’s ethics committee for the Institute of Psychiatry, Psychology and Neuroscience PNM/09/10–104. Only DZ co-twins with complete data were included in this study.

### Phenotypic Data

#### Height

Self-reported height was assessed at the average age of 22.1 (SD = 0.86) in 1,463 twin pairs.

#### Body Mass Index (BMI)

BMI was calculated using self-reported weight in kg and height in meters $\left(kg/m^{2}\right)$ at age 22.1 (SD = 0.86) in 1,353 twin pairs.

#### Self-Rated Health

Twins rated their health on the reduced RAND Short-Form Health Survey.^41^ Individuals scored their health on a five-point Likert scale for five questions such as “In general, would you say your health is”? (“Poor” to “Excellent”), or “I am as healthy as anybody I know” (“Strongly Disagree” to “Strongly Agree”). Data were available on 1,494 twin pairs at age 22.1 (SD = 0.86).

#### Intelligence

At age 11.4 (SD = 0.65), twins were assessed on their non-verbal abilities (Raven’s Standard Progressive Matrices;^42^ WISC-III-UK Picture Completion^43^) and on their verbal abilities (WISC-III-PI Vocabulary Multiple-Choice;^44^ WISC-III-PI Information Multiple-Choice^44^). A composite variable was calculated as the arithmetic mean of the z-standardized scales for 1,569 twin pairs.

#### Educational Achievement

Results for standardized tests taken at the end of compulsory education in the United Kingdom (General Certificate of Secondary Education; GCSE) were obtained for twins at age 16.3 (SD = 0.29) via self-report. Grades were coded from 4 (G; the minimum pass grade) to 11 (A^∗^; the highest possible grade). Self-reported GCSE grades in TEDS highly correlate with grades obtained for a subsample of individuals from the National Pupil Database (*r* = 0.98 for English, *r* = 0.99 for mathematics, *r* > 0.95 for all sciences).^31^ A composite was calculated as the arithmetic mean of the compulsory core subjects—Maths, English, and Science—for 2,366 twin pairs.

#### Neuroticism

At age 16.5 (SD = 0.27), twins were assessed on their Big Five personality traits on a five-point Likert scale.^45^ For this study, we used the six neuroticism items (e.g., anxiousness, vulnerability) to form a composite score by taking the arithmetic mean for 789 twin pairs.

#### Attention-Deficit Hyperactivity Disorder (ADHD) Symptoms

At age 11.5 (SD = 0.69) and 16.3 (SD = 0.69), parents reported on twins’ ADHD symptoms via the Strength and Difficulties Questionnaire^46^ hyperactivity subscale (three-point Likert scale) and the Conners’ rating scales (CPRS-R; four-point Likert scale)^47^ on hyperactivity and inattention. Although self-report ratings were available, it has been shown that informant-based ratings are more reflective of objective measures of ADHD symptoms.^48^ A composite score was created as the arithmetic mean of the sex and age z-standardized scales. Where ratings were available at one assessment only, this value was used to maximize sample size, leading to a sample of 2,469 twin pairs.

#### Schizophrenia Symptoms

At age 22.7 (SD = 0.85), paranoia and hallucinations were assessed through self-reported ratings on the Specific Psychotic Experiences Questionnaire (SPEQ; six-point Likert scale),^49^, ^50^ and parent-reported negative symptoms using the Scale for the Assessment of Negative Symptoms (SANS; four-point Likert scale).^51^ Data were available for 1,140 twin pairs.

#### Family Socio-economic Status (SES)

This measure was calculated as the mean of the z-standardized maternal age at birth of the first child, maternal and paternal highest education level (coded from 1 = “no qualifications” to 8 = “postgraduate qualifications”), and maternal and paternal occupation (coded from 1 = “Other Occupations – dockers, porters, labourers,…” to 9 = “Managers and Administrators”). These measures were assessed at first contact at age 1.8 (SD = 1.13). Data were available for 2,962 twin pairs.

Measures were selected based on largest sample sizes available, and ages at phenotype assessment matching most closely the ages of GWA study samples to maximize predictive power. None of the measures were significantly associated with birth order, but most showed sex and age differences (see Table S1) and were therefore adjusted for these effects using the regression method, and z-standardized residuals (mean = 0, SD = 1) were used for all subsequent analyses.

### Genotypic Data

Two different genotyping platforms were used because genotyping was undertaken in two separate waves, 5 years apart. AffymetrixGeneChip 6.0 SNP arrays were used to genotype 3,665 individuals. Additionally, 8,122 individuals (including 3,607 DZ co-twin samples) were genotyped on Illumina HumanOmniExpressExome-8v1.2 arrays. After quality control, 635,269 SNPs remained for AffymetrixGeneChip 6.0 genotypes, and 559,772 SNPs for HumanOmniExpressExome genotypes.

Genotypes from the two platforms were separately phased and imputed into the Haplotype Reference Consortium (release 1.1) through the Sanger Imputation Service^52^ before merging. Genotypes from a total of 10,346 samples (including 3,320 DZ twin pairs and 7,026 unrelated individuals) passed quality control, including 3,057 individuals genotyped on Affymetrix and 7,289 individuals genotyped on Illumina. The identity-by-descent (IBD) between individuals was < 0.05 for 99.5% in the sample excluding the DZ co-twins (range = 0.00 – 0.12) and ranged between 0.36 and 0.62 for the DZ twin pairs (mean = 0.49). The final data contained 7,363,646 genotyped or well-imputed SNPs (for full genotype processing and quality control details, see Selzam et al.^53^). To ease high computational demands of the software that generates polygenic scores, we further excluded SNPs with info < 1, leaving 515,000 SNPs for analysis.

We performed principal component analysis on a subset of 39,353 common (MAF > 5%), perfectly imputed (info = 1) autosomal SNPs, after stringent pruning to remove markers in linkage disequilibrium (r^2^ > 0.1) and excluding high linkage disequilibrium genomic regions to ensure that only genome-wide effects were detected.

### Polygenic Scores

We calculated polygenic scores, which are the SNP effect size weighted sums of the number of trait-associated alleles, based on summary statistics for the largest GWA studies available for key developmental outcomes, including height,^54^ body mass index (BMI),^54^ self-rated health,^55^ intelligence,^56^ educational attainment,^6^ neuroticism,^57^ ADHD,^58^ and schizophrenia.^59^ These GWA studies were selected because their respective GPS yield the highest predictive accuracy within their trait category (details about the studies, reported SNP heritabilities, and GPS predictions can be found in Table S2). To calculate the polygenic scores, we used the software LDpred^60^ which re-weights the SNP effect sizes based on a prior on the effect size and the LD in the sample. Here, we applied a prior on the fraction of causal markers of 1 for all analyses, based on the assumption that all genetic markers contribute to trait development (see Supplemental Material and Methods for details on polygenic score calculation). All polygenic scores were statistically adjusted for the first ten principal components, chip and plate using the regression method, and were z-standardized (mean = 0, SD = 1).

### Statistical Analysis

#### Mixed-Effects Modeling

We applied a random intercept mixed-effects model on DZ data, including two fixed effects to separate the total effect between the polygenic score predictor and the outcome into within- and between-family effects:^61^

(Equation 1) $$Y_{ij}=\alpha_{0}+\beta_{W}\left(GPS_{ij}-{\bar{GPS}}_{j}\right)+\beta_{B}{\bar{GPS}}_{j}+\gamma_{j}+\varepsilon_{ij},$$

where *Y* denotes the outcome and GPS the polygenic score, *i* = {1,2} corresponds to the individual twins that are clustered within family *j*, and$\;\bar{GPS}$ refers to the mean GPS value in family *j*. The *i*^th^ value represents birth order, where twin 1 is the elder twin. The notation $\alpha_{0}$ represents the intercept and $\gamma_{j}$ the random effect with $\gamma_{j}\;∼\;N\left(0,\sigma_{\gamma }^{2}\right)$, which corresponds to a change in the intercept for both twins in family *j*, and $\varepsilon_{ij}$ with $\varepsilon_{ij}\;∼\;N\left(0,\sigma_{\varepsilon }^{2}\right)$, which denotes the independent random error for each individual *i* in family *j*. The between-family effect $\beta_{B}$ represents the expected change in the outcome *Y* given a one unit change in the family GPS average, and the within-family effect $\beta_{W}$ represents the expected change given a one unit change in the difference between the individual GPS and the family average GPS. By including both $\beta_{W}$ and $\beta_{B}$ in the same model, the individual estimates are adjusted for, and independent of, the effect of the other estimate. The random effect term $\sigma_{\gamma }^{2}$, which estimates the difference between each group intercept $\gamma_{j}$ and the overall intercept $\alpha_{0}$, accounts for the residual structure in the data corresponding to all unaccounted familial factors (both genetic and environmental) that contribute to the trait similarity of the twins.^61^, ^62^

The use of a mixed-effects model is only justified if co-twins within a family correlate in the outcome, which can be estimated through the intraclass correlation coefficient (ICC). The ICC is the ratio of the between-family (i.e., random intercept) variance over the total variance and is an estimate of how much of the total variation in the outcome is accounted for by family:

(Equation 2) $$Cor\left(Y_{1j},Y_{2j}\right)=\;\frac{\sigma_{\gamma }^{2}}{\left(\sigma_{\gamma }^{2}+\sigma_{\varepsilon }^{2}\right)},$$

where $\sigma_{\gamma }^{2}$ is the covariance between the family variable, in this case family ID, and the outcome, and $\sigma_{\varepsilon }^{2}$ indicates the residual variance capturing within-twin pair differences. The total effect of the relationship between GPS and outcome is the ICC weighted sum of the within- and between-family effects:^62^

(Equation 3) $$Total\;effect=\;\beta_{W}\left(1-ICC\right)+\;\beta_{B\;}ICC.$$

It follows from Equation 3 that the total effect ranges between $\beta_{W}$ and $\beta_{B}$. If the relationship between GPS and outcome is mostly due to individual-level variation, the ICC approximates 0 and the total effect will be close to $\beta_{W}$. In contrast, if the association is mostly due to family effects, the ICC approximates 1 and the total effect will be close to $\beta_{B}$.^62^ To calculate the total effect, we used ICC estimates adjusted for the fixed effects described in Equation 1.

Performing a regression corresponding to Equation 1, we estimated the $\beta_{W}$ and $\beta_{B}$ parameters using each of the eight polygenic scores in turn as predictors of each of the eight measured outcomes. To estimate potential SES effects, we repeated these analyses including the SES composite as a covariate in the model (these latter analyses were not preregistered). For the fixed effects, we calculated 95% bootstrap percentile intervals. These were based on 10,000 bootstrap samples with random resampling of DZ twin pairs with replacement.

To empirically test the statistical difference between $\beta_{W}$ and $\beta_{B}$, we divided the difference between the fixed effect coefficients by the standard deviation of the sampling distribution of the estimate differences.^63^, ^64^ We also applied this approach to statistically test the significance of the difference between the $\beta_{B}$ coefficients before and after the inclusion of family SES in Equation 1. To evaluate the effect size change between the coefficients, we calculated the beta differences with 95% bootstrap percentile intervals, as well as the percentage change (e.g., $\left(\left(\beta_{B}-\beta_{W}\right)/\beta_{B}\right)$).

#### Quantile Analysis of Within-DZ Pair Differences

To illustrate the extent to which within-DZ pair GPS differences result in differences in developmental outcomes, we performed quantile analysis. First, we generated twin-GPS difference scores by subtracting the twin 2 score from the twin 1 score, and then split this variable into ten equal quantiles based on absolute GPS differences, ranging from the lowest to the highest GPS differences. Birth order did not explain any statistically significant amount of variance (Table S1), so no randomization of twin order was required. We tested mean differences in outcome variables between individuals in the lowest and highest decile. We performed quantile analysis on variables with scales that are easily interpretable: that is, BMI, height, intelligence, and educational achievement. For this purpose, the z-standardized and cleaned variables were transformed back to their original scale, and intelligence values were scaled to have a mean of 100 and a standard deviation of 15.

#### Multiple Testing Correction

Multiple testing correction of the α significance threshold was performed using the Benjamini Hochberg false discovery rate (FDR) adjustment.^65^ In contrast to more conservative corrections, this method has higher statistical power to detect true positives while controlling for false positives. Based on an α threshold of 0.05, the corrected α in this study was 0.01, defined as the maximum raw p value that is smaller than or equal to the FDR critical value $\left(p_{raw}\leq \left(rank\;of\;p_{raw}/total\;number\;of\;p_{raw}\;values\right)\times \text{α}\right).$

#### Sensitivity Analyses

We performed additional, non-pre-registered sensitivity analyses to evaluate the robustness of our findings. Mixed-effects models were run separately for same-sex and for opposite-sex twin pairs (for twin pair *N*, see Table S3), as well as for twin pairs where both twins were genotyped on the OEE chip, and twin pairs where one twin was genotyped on OEE and the other twin genotyped on Affymetrix (for twin pair *N*, see Table S4). Analyses were also performed using GPSs that were constructed applying a causal fraction of 0.1.

To control for any unaccounted relatedness between families, we estimated the fixed effects including a SNP-kinship matrix as random effect. Here, Equation 3 becomes $y=\;\alpha +\;\beta_{W}\left(GPS_{ij}-{\bar{GPS}}_{j}\right)+\beta_{B}{\bar{GPS}}_{j}+g+\varepsilon$, where *g* is the random effect with $g\;∼\;N\left(0,A\sigma_{g}^{2}\right)$ and *A* being a genetic relationship matrix between individuals. A pairwise genetic relationship between individual *m* and *n* is estimated as $A_{mn}=1/N\;\sum_{i=1}^{N}\left(x_{im}-\;2p_{i}\right)\left(x_{in}-\;2p_{i}\right)/2p_{i}\left(1-\;p_{i}\right),$ where *N* is the number of SNPs, *x*_*im*_ is the number of copies of the reference allele for the *i*^th^ SNP of the *m*^th^ individual and *p*_*i*_ is the reference allele frequency. These analyses were performed using the GCTA software (v.1.90.0).^66^

Due to the large study population of the UK Biobank, there may be relatedness between this sample and the UK target sample TEDS. The UK Biobank sample was included in the GWA meta-analysis of height, BMI, and educational attainment, and relatedness between discovery and target sample could lead to GPS prediction estimate inflation in the target sample.^67^, ^68^ We therefore calculated an additional set of height and BMI polygenic scores based on GWA meta-analyses published before UK Biobank data became available.^69^, ^70^ We also calculated an additional GPS for educational attainment based on a GWA analysis that had all British cohorts removed.^6^ While this rules out discovery and target sample relatedness, it also controls for effect size inflation due to population stratification.

## Results

Phenotypic resemblance between DZ twins within a family varied across traits, with Pearson’s correlation coefficients ranging from 0.10 to 0.59 (Figure S1, and Table S5 for ICCs). Twins were least alike in their neuroticism levels and self-rated health, and most alike in their height, IQ, and educational achievement. Within-twin pair polygenic score correlations were close to expectations (range *r* = 0.49–0.57), as the expected shared additive genetic variance between siblings is 50% of the total additive genetic variance based on quantitative genetic theory.^22^ Given the 95% confidence intervals of the within-twin pair correlations (Figure S1), there was a significant difference from the expected correlation coefficient of 0.50 for the self-rated health GPS (*r* = 0.53), the IQ GPS (*r* = 0.54), and the educational attainment GPS (*r* = 0.57), indicating assortative mating for these traits.

### Within-Family Polygenic Score Predictions

**Figure 1:**
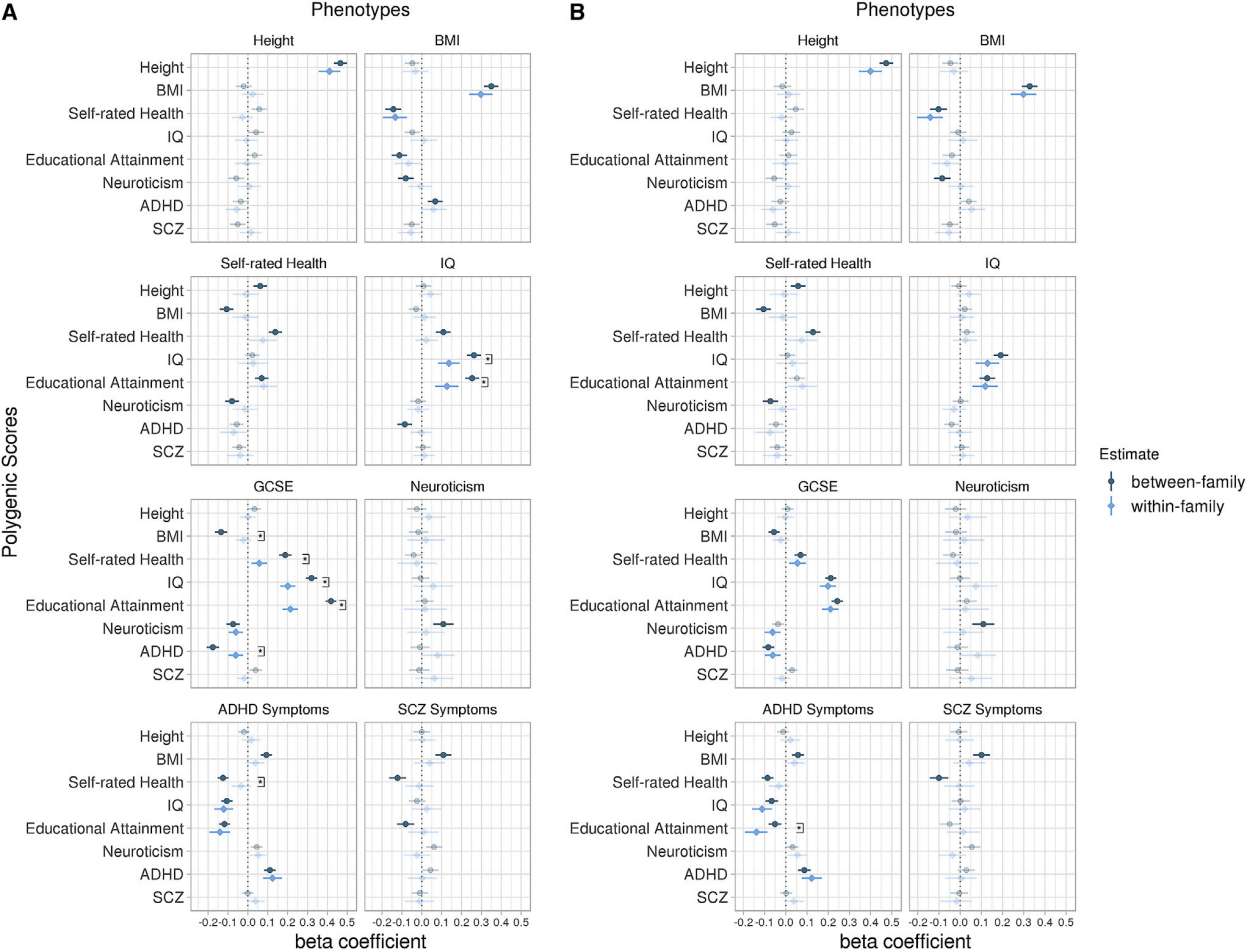
Within- and Between-Family Prediction Estimates of Eight Outcomes using Eight Genome-wide Polygenic Scores Findings before (A) and after (B) statistical correction for family socio-economic status (SES). The genome-wide polygenic scores (GPS) are presented on the y axis, predicting each of the eight phenotypic traits. Error bars are 95% bootstrap percentile intervals based on 10,000 bootstrap samples (random resampling of DZ twin pairs with replacement). Opaque estimates indicate statistical significance at the false discovery rate corrected threshold of p < 0.01. Brackets indicate a significant difference between within- and between-family prediction estimates. Significant differences are only shown where at least one of the estimates is statistically significant at the false discovery rate corrected threshold of p < 0.01 (for all prediction estimates and p values, see Tables S6 and S7). The dotted line represents a beta coefficient of zero. BMI, body mass index; IQ, intelligence; GCSE, general certificate of secondary education (educational achievement); ADHD, attention-deficit/hyperactivity disorder; SCZ, schizophrenia.

Figure 1A depicts the within- and between-family polygenic score prediction estimates of the eight outcomes from the mixed-effects model analyses. Within-family target-trait predictions were statistically significant for height, BMI, intelligence, educational achievement, and ADHD symptoms, indicating that polygenic variation within twin pairs was related to these outcome differences. Specifically, phenotypic differences in height were significantly positively correlated with height GPS twin differences (*β* = 0.41, p = 5.72e^−53^) and differences in BMI were significantly correlated with BMI GPS differences (*β* = 0.30, p = 1.76e^−21^) such that twins with a higher height GPS and BMI GPS were taller and heavier than their co-twin, respectively. IQ GPS differences predicted intelligence differences (*β* = 0.14, p = 1.32e^−6^) and EA GPS differences were significantly associated with GCSE grade differences (*β* = 0.21, p = 2.22e^−26^), indicating that those twins with a higher GPS also scored higher on intelligence measures and in their GCSE tests than their co-twin. For behavior problems, twins with higher ADHD GPS had higher phenotypic ADHD symptoms than their co-twins (*β* = 0.12, p = 1.50e^−7^).

We also investigated cross-trait relationships (Figure 1A). For example, self-rated health GPS differences were negatively correlated with differences in BMI, such that twins with a higher self-rated health GPS had a lower BMI (*β* = −0.13, p = 3.56e^−5^). EA GPS differences significantly related to phenotypic intelligence differences (*β* = 0.13, p = 2.15e^−5^), and IQ GPS predicted GCSE grade differences (*β* = 0.20, p = 7.24e^−25^), suggesting that those with higher GPSs also had higher IQ and GCSE grades than their co-twin. GCSE grade differences were also negatively predicted by ADHD GPS twin differences (*β* = −0.07, p = 2.20e^−4^), indicating that twins with a higher ADHD GPS obtain lower GCSE results. Notably, IQ GPS differences (*β* = −0.12, p = 6.38e^−7^) and EA GPS differences (*β* = −0.14, p = 3.09e^−8^) were just as predictive of ADHD symptoms as the ADHD GPS itself, and the direction of effect sizes indicates that the twin with a higher GPS had lower ADHD symptoms than their co-twin (all prediction estimates and total effects are presented in Table S6).

### Comparing Within-Family and Between-Family Polygenic Score Prediction

By simultaneously and independently estimating within- and between-family GPS predictions, it was possible to compare these estimates. Between-family estimates (Figure 1A) are mostly consistent with GPS correlations reported for unrelated individuals (Table S2). Figure 1A also shows that between-family associations are generally greater than within-family associations. Significant associations were found for 46.9% of the between-family associations and only 20.3% for within-family associations. On average, magnitudes of within-family associations were almost half (44.1% reduction) that compared to significant between-family estimates (for all prediction estimates, beta difference values and their 95% confidence intervals, and significance of differences, see Table S6).

Notably, significant differences in associations within and between families for polygenic scores predicting their target traits were almost exclusively found for IQ and educational achievement (Figure 1A). The within-family prediction was significantly lower than between-family prediction for both IQ (p = 6.27e^−4^, Δ = 48.0%) and GCSE grades (p = 8.45e^−14^, Δ = 48.9%). Despite not reaching statistical significance, we also observed attenuation of the within-family prediction relative to the between-family prediction for height (Δ = 11.8%), BMI (Δ = 15.1%), self-rated health (Δ = 45.2%), and neuroticism (Δ = 80.4%).

Also, for cross-trait associations, differences in within- and between-family polygenic score predictions were most pronounced for IQ and educational achievement. For IQ, there were significant differences for the EA GPS (p = 7.57e^−4^, Δ = 50.1%). For educational achievement, there were significant differences for the BMI GPS (p = 8.10e^−5^, Δ = 83.3%), the self-rated health GPS (p = 4.60e^−6^, Δ = 69.5%), the IQ GPS (p = 1.79e^−5^, Δ = 37.2%), and the ADHD GPS (p = 4.95e^−5^, Δ = 65.4%). In addition, there was a significant difference in within- and between-family prediction for the self-rated health GPS (p = 4.00e^−3^, Δ = 71.7%) predicting ADHD symptoms. Although not significant, effect size attenuations were also sizeable for other cross-trait predictions, such as for the neuroticism GPS predicting BMI (Δ = 91.2%) or the self-rated health GPS predicting schizophrenia symptoms (Δ = 90.1%) (Table S6). However, for these comparisons, between-family coefficients may not be as reliable as the between-family coefficients that showed a significant difference to their within-family estimate, as estimates were considerably smaller to begin with.

The finding that polygenic score prediction estimates of our measured traits are substantially smaller within families suggests that the corresponding between-family associations are mediated by some combination of family-specific (i.e., shared family) effects, population stratification, and potentially assortative mating. Family SES, which is the same for members of a family, is a predictor not only of educational achievement and IQ but also physical and mental health outcomes. Therefore, we repeated our analyses including family SES as a covariate in the model to interrogate its role in between-family GPS prediction. As noted above, this analysis was not pre-registered. As shown in Figure 1B, between-family predictions were greatly reduced and magnitudes approached those of within-family prediction estimates, which did not change (because any shared family effects are already controlled for in within-family estimates; for all prediction estimates, beta difference values and their 95% confidence intervals, and significance of differences, see Table S7).

Formal testing of the between-family estimate differences before and after correcting for SES indicated significant differences only for cognitive traits (Table S8, Figure S2). For example, there was an average attenuation of 60.9% across the within- and between-family comparisons for the GPSs that showed a statistically significant difference in their prediction of GCSE grades, which was reduced to 25.8% after accounting for SES. Although this is a substantial attenuation, these findings show that family SES does not account for all of the observed differences.

We performed additional contrasts, controlling for the SES components parental education and parental occupation separately in an attempt to identify more specific potential sources of prGE. For GCSE grades and IQ, between-family beta coefficients showed greater attenuation when controlling for parental education in comparison with parental occupation (Tables S9 and S10, Figure S3). However, only for the educational attainment GPS predicting GCSE grades was the difference between the attenuation due to parental education (Δ = 18.9%) and parental occupation (Δ = 37.7%) statistically significant (p = 6.40e^−3^) (Table S11), indicating that parental education may present a stronger prGE effect.

As a further set of analyses, we applied a multiple regression approach to predict family SES using the within- and between-family estimates of the eight GPSs. Family SES acts as a control trait as there should be no direct genetic effects from the offspring to family SES, as indicated by the within-family effect. Results confirmed that all within-family beta coefficients were zero, while between-family estimates were related to family SES (Table S12).

Sensitivity analyses (not pre-registered) were performed by repeating all analyses separately for same-sex and opposite-sex twins (Tables S3, S13, S14, S15, and S16, and Figures S4 and S5), and for twin pairs grouped by genotyping chip (Tables S4, S17, S18, S19, and S20, and Figures S6 and S7). In addition, we estimated the fixed effects using a SNP-kinship matrix as a random effect to control for any unaccounted between-family relatedness (Table S21 and Figure S8). For the different sets of sensitivity analyses described, no substantial deviations from the results using the combined sample were found.

We also repeated analyses using GPSs that were calculated based on a fraction of causal markers of 0.1 (Tables S22 and S23, and Figure S9), and using GPSs that had the UK Biobank sample (height; BMI) or all British samples (educational attainment) removed at the GWA analysis stage (Table S24 and Figure S10). Although prediction estimates were smaller in some cases likely due to reduced power, the pattern of within- versus between-family effect size changes remained unchanged as indicated by the mostly overlapping 95% confidence intervals of the beta difference values.

### Quantile Analysis

**Figure 2:**
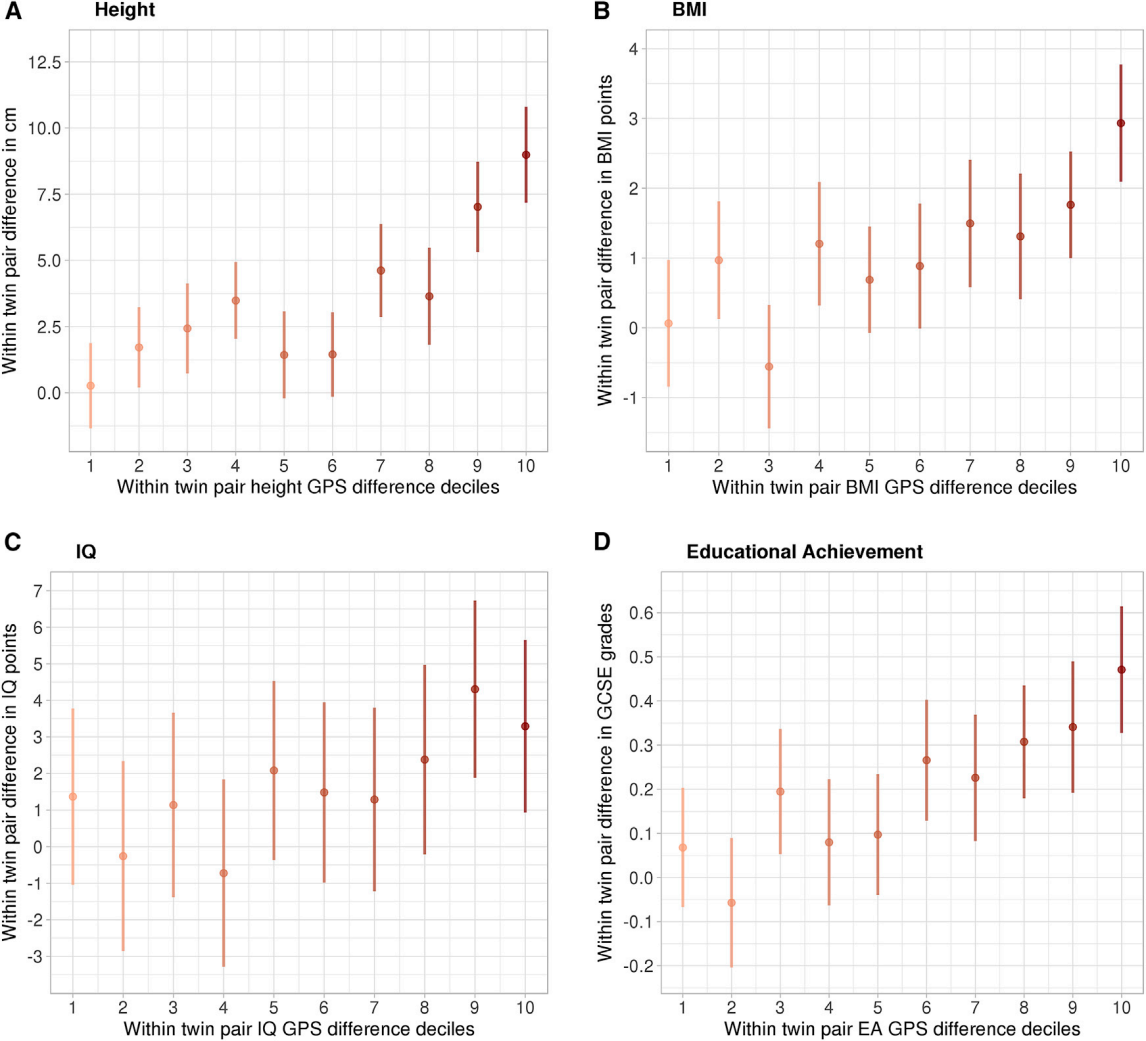
The Relationship between Absolute Dizygotic (DZ) Twin Pair Polygenic Score Decile Differences and Trait Outcome Differences Lower deciles represent small absolute genome-wide polygenic score (GPS) differences and higher deciles represent large GPS differences between DZ co-twins. Error bars indicate 95% confidence intervals. Each GPS decile included the following numbers of twin pairs: height = 146; BMI = 135; IQ = 157; GCSE = 236. Regression through origin analysis (fixed intercept of zero) using the continuous GPS difference values to predict outcome differences were significant for height (*Β* = 4.42, p = 3.73e^−53^, R^2^ = 0.148), BMI (*Β* = 1.34, p = 1.73e^−21^, R^2^ = 0.064), IQ (*Β* = 2.1, p = 4.53e^−7^, R^2^ = 0.015), and GCSE grades (*Β* = 0.26, p = 3.04e^−26^, R^2^ = 0.046).

To illustrate within-family differences further, quantile analysis demonstrated how within-family polygenic score differences related to differences in height, BMI, IQ, and GCSE grades (Figure 2). There was an 8.7 cm height mean difference (p = 1.28e^−11^) between the lowest absolute difference decile versus the highest difference decile. For BMI, the difference was 2.9 BMI points (p = 8.33e^−6^) between the lowest and the highest absolute GPS difference deciles. Mean GCSE grade differences (0.40) were also statistically significant (p = 7.13e^−5^) when comparing the lowest and the highest absolute GPS difference deciles. In contrast, IQ point differences (1.9 points) were not statistically different (p = 0.26) between the lowest and the highest absolute GPS difference quantiles (for trait and GPS means at each difference decile, see Table S25).

## Discussion

Polygenic score prediction of complex traits is now a common approach in genomics research, but the potential pathways by which polygenic score variation predicts phenotypic variation remain largely unexplored. In this study, we contrasted within- and between-family polygenic prediction estimates to quantify the extent to which environmentally mediated genetic effects (i.e., passive genotype-environment correlation) are picked up in polygenic score analyses. By systematically performing target- and cross-trait analyses across eight life outcomes using eight corresponding GPS, we found evidence that prGE might be a mechanism explaining a considerable proportion of the GPS prediction in cognitive traits (intelligence and educational achievement), but not as much for non-cognitive traits. We also found that for between-family GPS predictions of cognitive traits—but, again, not as much for other traits—family SES is likely to be the major source of prGE.

For the prediction of IQ and educational achievement, within-family estimates were on average 60% smaller than between-family estimates. The within- versus between-family attenuation for the EA GPS prediction was 49%, which is close to the 40% estimate in GWA study effect sizes for years of education^6^ and the 55% estimate using the same EA GPS in a different target sample.^29^ These findings highlight the influence of prGE in the development of IQ and educational achievement and demonstrate the extent to which between-family GPS prediction may be partly driven by prGE effects. Results from our study are also in line with adoption studies showing evidence of between-family prGE in that correlations between home environment and children’s IQ is twice as great in non-adoptive families than in adoptive families.^71^ Our findings are compatible with recent research on *genetic nurture*, using non-transmitted alleles from parental genotypes to assess prGE^20^, ^21^ in terms of GPS target trait prediction of educational achievement and anthropometric traits. Our findings also extend to cross-trait associations using a wide range of GPSs. Contrary to our prediction that within- and between-family EA GPS associations would be significantly different across many associated life outcomes, results from cross-trait analysis suggest that within- and between-family predictions were only significantly different across a range of GPS for the prediction of cognitive outcomes.

A possible explanation for these results is that IQ and educational achievement show more shared environmental influences (24% and 27%, respectively) relative to other traits used in this study such as height (10%), BMI (10%), ADHD (2%), or schizophrenia (0%), as estimated through a large twin study meta-analysis.^72^ The type of rGE that we assessed in this study—defined as the exposure to a family environment that is correlated with both parental and offspring genotypes and which contributes to sibling similarity in their outcomes—is absorbed by the shared environment variance component (C) in classical twin analyses.^73^ Therefore, it may be more likely that genetic effects related to cognitive traits as estimated through GWA studies partly contain prGE effects—in contrast to other traits tested in our study—because the shared environmental component is larger to begin with for cognitive traits. In TEDS, the C component for the same IQ and educational achievement measures used in this study were estimated around 35%^74^ and 29%,^75^ respectively.

As known from the existing literature, family SES is strongly genetically correlated with offspring cognitive traits,^8^, ^37^, ^38^ rendering it a likely source of prGE. Indeed, our results showed that between-family effects were considerably more similar in magnitude to within-family effects when holding SES constant, suggesting that SES is a source of the majority of the within-between discrepancy, rather than residual population stratification or assortative mating. When controlling for parental education and parental occupation separately, we found that between-family effect sizes were closer to within-family coefficients for parental education than for occupation. However, this difference was significant only for the educational attainment GPS predicting GCSE grades, suggesting that parental education is likely a stronger source of prGE than parental occupation influencing offspring educational achievement.

Despite the sizeable attenuations after controlling for family SES, we still observed some effect size differences when comparing within- and between-family coefficients. For example, there was still a 32.5% difference for the IQ GPS predicting IQ and a 13.4% difference for the educational attainment GPS predicting GCSE scores. The within-twin pair correlations for these GPS indicated assortative mating, which could explain some of this remaining discrepancy. Indeed, previous research on genetic nurture indicated that a small proportion of the direct genetic effect of the educational attainment polygenic score predicting educational attainment captures assortative mating-related effects.^20^ The same research also showed genetic nurture effects between siblings using the educational attainment GPS.^20^ Such effects may further contribute to the within-family effect attenuation, potentially accounting for some of the residual difference after controlling for family SES.

The results showed that more distantly related GPS captured considerable prGE effects in cross-trait GPS predictions of cognitive traits. For instance, within-family effect sizes for the ADHD GPS predicting educational achievement were significantly smaller (65% reduction), in contrast to the ADHD GPS predicting ADHD symptoms, where no difference was detected. This suggests that the GWAS for ADHD captures genetic variation that is correlated with aspects of the family environment that contribute to the co-development of ADHD symptoms and educational achievement, although it is unclear why these effects do not appear to contribute to the development of ADHD symptoms themselves.

It is important to go beyond GPS predictions of traits in unrelated individuals to consider prGE mechanisms by comparing within- and between-family predictions in order to explain the sources of predictions in polygenic score analysis. However, finding between-family prGE does not diminish the usefulness of GPS predictions for cognitive traits in unrelated individuals, because these prGE effects help maximize the prediction of trait variance. Although within-family genetic effects do not include prGE effects due to between-family factors such as SES, within-family genetic effects are not free of *all* kinds of rGE, as demonstrated by twin studies showing that correlations between putative measures of the environment and children’s specific outcomes are genetically influenced.^71^ Within-family GPS prediction estimates can be interpreted as direct genetic effects in the sense that they stem from the individual level and not the family level. Children select, modify, and create experiences (active rGE) or evoke responses in their environment (evocative rGE) that are correlated with their genetic propensities. Therefore, within-family genetic differences can relate to trait differences through active or evocative rGE pathways but are free of any passive rGE effects.

### Implications

The results from this study have three important implications for the interpretation of the existing polygenic score literature as well as for future genetic research. First, the finding that between-family predictions pick up effects due to prGE mostly and substantially in cognitive traits is informative for causal inference studies that use designs such as Mendelian randomization.^76^, ^77^ Here, a genetic instrument that is related to a predictor (in the form of a single genetic marker or GPS) is used to assess the causal relationship between the predictor and an outcome. At a population level, genotypes are not inherited randomly: individuals with particular genotypes are not born into environmental conditions at chance. If family environment is associated with the genetic instrument as well as the predictor and the outcome, this opens a backdoor path whereby predictor and outcome are related through the prGE mechanisms.^19^ This could lead to an assumption violation, therefore biasing causal inference in between-family analysis. Only in a within-family design is it ensured that Mendelian randomization meets its assumptions because transmission of alleles is randomized at meiosis within families, and because prGE effects due to shared environment are held constant.^19^, ^26^, ^78^, ^79^ Although genetic data for siblings are often not available, our results provide a useful guideline for the GPS-outcome combinations that are unlikely to suffer from this assumption violation when applying designs such as Mendelian randomization to unrelated samples. For example, our results indicate that caution should be warranted due to prGE mechanisms if applying Mendelian randomization to cognitive traits, even if family SES is included as a confounder in the analyses as confounding effects might not be captured perfectly. In contrast, other traits such as BMI and ADHD (with the possible exclusion of the self-rated health GPS) should be less problematic, because within- and between-family effect sizes match closely, ruling out potential confounding due to prGE.

Second, our results provide evidence that location-related population stratification is not a large bias in GPS prediction of complex traits when controlling for genetic principal components in samples from white European backgrounds. As it has been shown that the GPS prediction of height is affected by population stratification,^80^ we also find an attenuation of around 12% of the within-family coefficient, which is by necessity free of population stratification since stratification is constant within a family. When we performed our analyses using a GPS for height based on a discovery sample that did not include UK Biobank, the attenuation decreased to 5%. This may indicate that the inclusion of a large discovery sample genetically similar to the target sample could have resulted in a GPS that is more strongly confounded by population stratification—although it is noteworthy that the 95% confidence intervals of the beta difference values overlap for the two height GPSs. For those traits where within- and between-family estimate differences were large and significant, differences were greatly reduced after accounting for SES, indicating that SES was the main source of the discrepancy, as opposed to location-related population stratification. Our additional analyses using a GPS based on GWA analysis that had all British samples removed did not show less attenuation, which would be expected if population stratification strongly influenced GPS prediction.

Third, our study illustrates the usefulness of obtaining genotypic data on family members, since it makes it possible to identify mechanisms of polygenic prediction. Our results demonstrate that by analyzing DZ co-twins’ genetic data jointly, prGE mechanisms due to shared environment (and in this case associated with SES) can be revealed.

### Limitations

There are some limitations to this study. The GWA studies used to generate the eight GPS for this study had different statistical power to discover genetic effect sizes due to sample size variations and different underlying genetic architectures of the GWA study traits. As a result, each of the eight GPSs were differently powered to detect target- and cross-trait associations, making it difficult to draw direct comparisons across the within- and between-family prediction effect sizes. Lack of power may also lead to an inability to detect small prGE effects that would become visible with (1) more powerful GPS and (2) the availability of larger DZ twin pair samples. However, we detected prGE effects in cross-trait analysis using the ADHD GPS, which is based on the smallest GWAS study sample (∼55,000 individuals), indicating that we had sufficient power to detect at least some of the prGE effects.

It is also possible that some important within- and between-family effect differences did not reach statistical significance due to insufficient statistical power. While the effect size differences in cognitive traits are large, it may be that effects due to prGE, population stratification and/or assortative mating are more subtle in other traits. Therefore, our study sample, which ranged between 789 and 2,469 DZ twin pairs, may have not had enough power to establish the statistical significance of small effect size differences. Notably, GPS predictions were generally small where no significant difference was found between large within- versus between-family effect size attenuations. With the availability of more powerful GPS in the future, it may be possible to detect such differences statistically.

Another limitation was that we did not have parental genotypes available to directly test the influence of non-transmitted parental alleles on offspring outcomes (genetic nurture).^20^ Although the within-family design used in this study accounts for the effects of both transmitted and non-transmitted parental alleles on offspring outcomes, it is not possible to disentangle these two sources of prGE. Future studies would benefit from incorporating parental and sibling genotypes to disentangle the prGE effects through the joint analysis of parental and sibling genotypes, which will shed light on how both non-transmitted parental and non-co-inherited sibling alleles contribute to trait development.

### Conclusion

This study provided strong evidence for prGE mechanisms in polygenic score prediction mainly for cognitive traits across a range of different polygenic scores. The implications of these findings for future studies depend on their aims. If maximizing trait prediction is the goal, the use of unrelated samples is valid even in the presence of prGE effects because these influences are informative nonetheless. However, if the goal is causal inference and explanation, a within-family genetic design is recommended to avoid prGE-related confounding. The increasing availability of genotypic data in relatives will become a crucial element in genetics research, allowing researchers to disentangle the mechanisms of polygenic prediction of complex human traits.

## Declaration of Interests

The authors declare no competing interests.

## Web Resources

OSF pre-registration link, https://osf.io/eq8ga/?view_only=768f42366d134ebeb50f5999763c3fce

## Supplemental Data

Document S1. Supplemental Material and Methods, Figures S1–S25, and Tables S1–S5, S12, S24, and S25

Table S6. Within- and Between-Family Prediction Estimates

Table S7. Within- and Between-Family Prediction Estimates after Accounting for Family Socio-Economic Status

Table S8. Comparison of Between-Family Coefficients before and after Controlling for Family Socio-Economic Status

Table S9. Within- and Between-Family Prediction Estimates after Accounting for Parental Education

Table S10. Within- and Between-Family Prediction Estimates after Accounting for Parental Occupation

Table S11. Comparison of Between-family Coefficients after Controlling for Parental Education and Parental Occupation

Table S13. Within- and Between-Family Prediction Estimates for Same-Sex Twin Pairs

Table S14. Within- and Between-Family Prediction Estimates after Accounting for Family Socio-Economic Status for Same-Sex Twin Pairs

Table S15. Within- and Between-Family Prediction Estimates for Opposite-Sex Twin Pairs

Table S16. Within- and Between-Family Prediction Estimates after Accounting for Family Socio-Economic Status for Opposite-Sex Twin Pairs

Table S17. Within- and Between-Family Prediction Estimates Based on Twin Pairs Genotyped on OEE Chip

Table S18. Within- and Between-Family Prediction Estimates after Accounting for Family Socio-Economic Status Based on Twin Pairs Genotyped on OEE Chip

Table S19. Within- and between-family prediction estimates based on twin pairs with one twin genotyped on the OEE chip and the co-twin genotyped on the Affymetrix chip

Table S20. Within- and Between-Family Prediction Estimates after Accounting for Family Socio-Economic Status Based on Twin Pairs with One Twin Genotyped on the OEE Chip and the Co-twin Genotyped on the Affymetrix Chip

Table S21. Within- and Between-Family Coefficients Estimated via a SNP-Kinship Matrix as Random Effect

Table S22. Within- and Between-Family Prediction Estimates via Polygenic Scores Calculated with Causal Fraction of Markers of 0.1

Table S23. Within- and Between-Family Prediction Estimates after Accounting for Family Socio-Economic Status via Polygenic Scores Calculated with Causal Fraction of Markers of 0.1

Document S2. Article plus Supplemental Information
