# Supplementary material for: Comparing Within- and Between-Family Polygenic Score Prediction
Source: Am J Hum Genet. 2019 Jul 11;105(2):351–63. doi: 10.1016/j.ajhg.2019.06.006 (PMC6698881; doi:10.1016/j.ajhg.2019.06.006)
Supplement: Document S1. Supplemental Material and Methods, Figures S1–S25, and Tables S1–S5, S12, S24, and S25 [file mmc1.pdf]

The American Journal of Human Genetics, Volume *105*

Supplemental Data

Comparing Within- and Between-Family Polygenic Score Prediction

Saskia Selzam, Stuart J. Ritchie, Jean-Baptiste Pingault, Chandra A. Reynolds, Paul F. O'Reilly, and Robert Plomin

Supplementary Figures

Figure S1. Within-twin pair Pearson's correlation coefficients.

Figure S2. Effect sizes and significance of differences comparing between-family coefficients before and after controlling for family socio-economic status

Figure S3. Within- and between-family prediction estimates accounting for separate measures of family socio-economic status

Figure S4. Within- and between-family prediction estimates for same-sex twin pairs

Figure S5. Within- and between-family prediction estimates for opposite-sex twin pairs

Figure S6. Within- and between-family prediction estimates based on twin pairs genotyped on OEE chip

Figure S7. Within- and between-family prediction estimates based on twin pairs genotyped on OEE chip and the co-twin genotyped on the Affymetrix chip

Figure S8. Within- and between-family coefficients estimated using a SNP-kinship matrix as random effect

Figure S9. Within- and between-family prediction estimates using polygenic scores calculated with causal fraction of markers of 0.1

Figure S10. Within- and between-family prediction estimates based on polygenic scores with UK Biobank or all UK discovery samples removed

Supplementary Tables

Table S1. Descriptive statistics, age and sex effects for phenotypes

Table S2. GWAS used for polygenic score calculation

Table S3. Intraclass coefficients for same-sex and opposite-sex twin pairs

Table S4. Intraclass coefficients for twin pairs split by genotyping chip

Table S5. Intraclass coefficients

Table S12. Within- and between-family prediction estimates of family socio-economic status

Table S24. Within- and between-family prediction estimates based on polygenic scores with UK Biobank or all UK discovery samples removed

Table S25 Phenotypic and polygenic score mean differences by polygenic score difference quantiles

Supplementary Methods

Methods S1. Polygenic score calculation

References

Supplementary Figures

Figure S1. Within-twin pair Pearson's correlation coefficients.

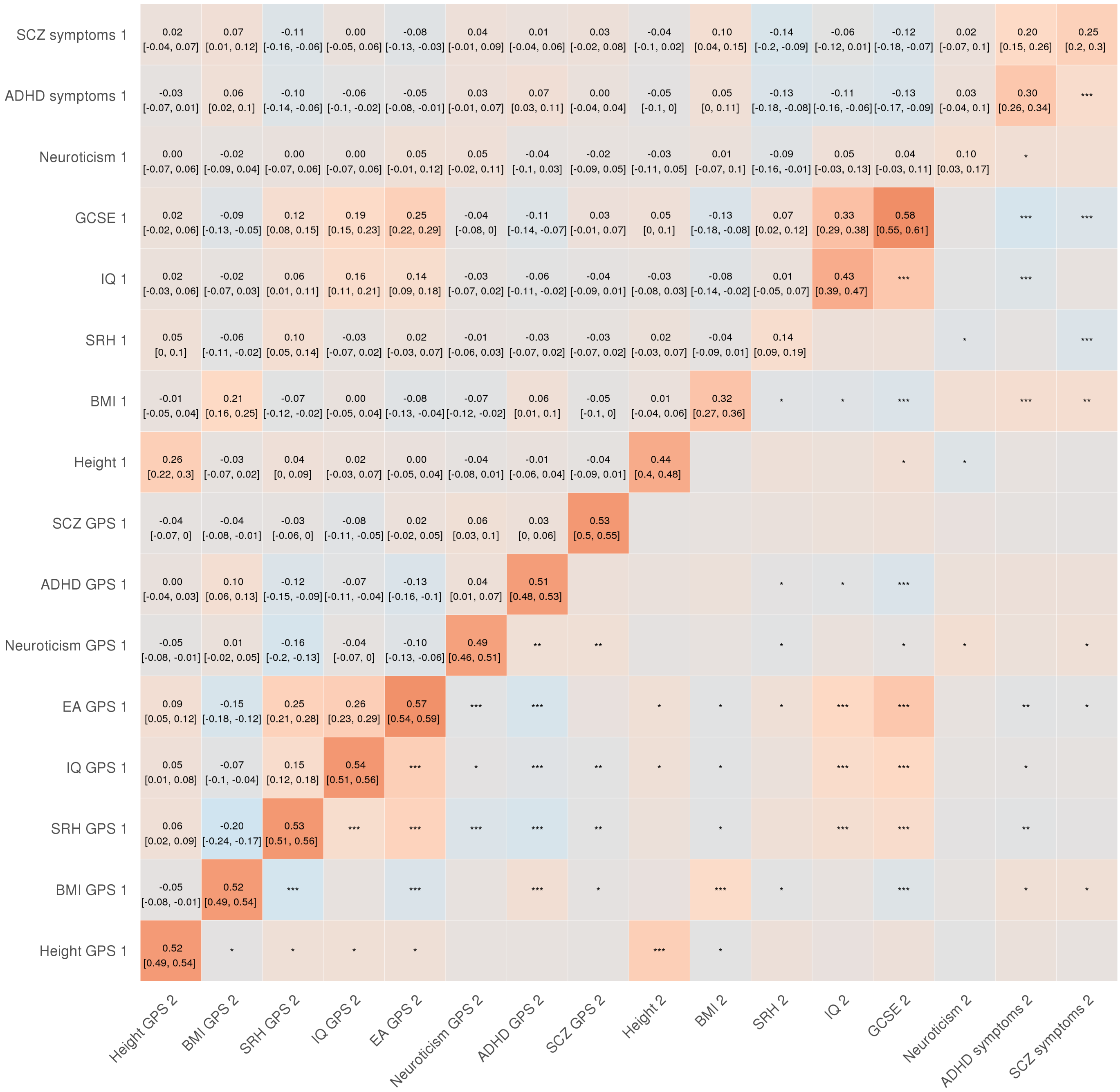

Note. BMI = Body Mass Index; IQ = Intelligence; GCSE = General Certificate of Secondary Education (educational achievement); ADHD = Attention-Deficit/Hyperactivity Disorder; SCZ = Schizophrenia; EA = Educational Attainment; SRH = Self-rated Health; 1 = Twin 1; 2 = Twin 2.

Figure S2. Effect sizes and significance of differences comparing between-family coefficients before and after controlling for family socio-economic status

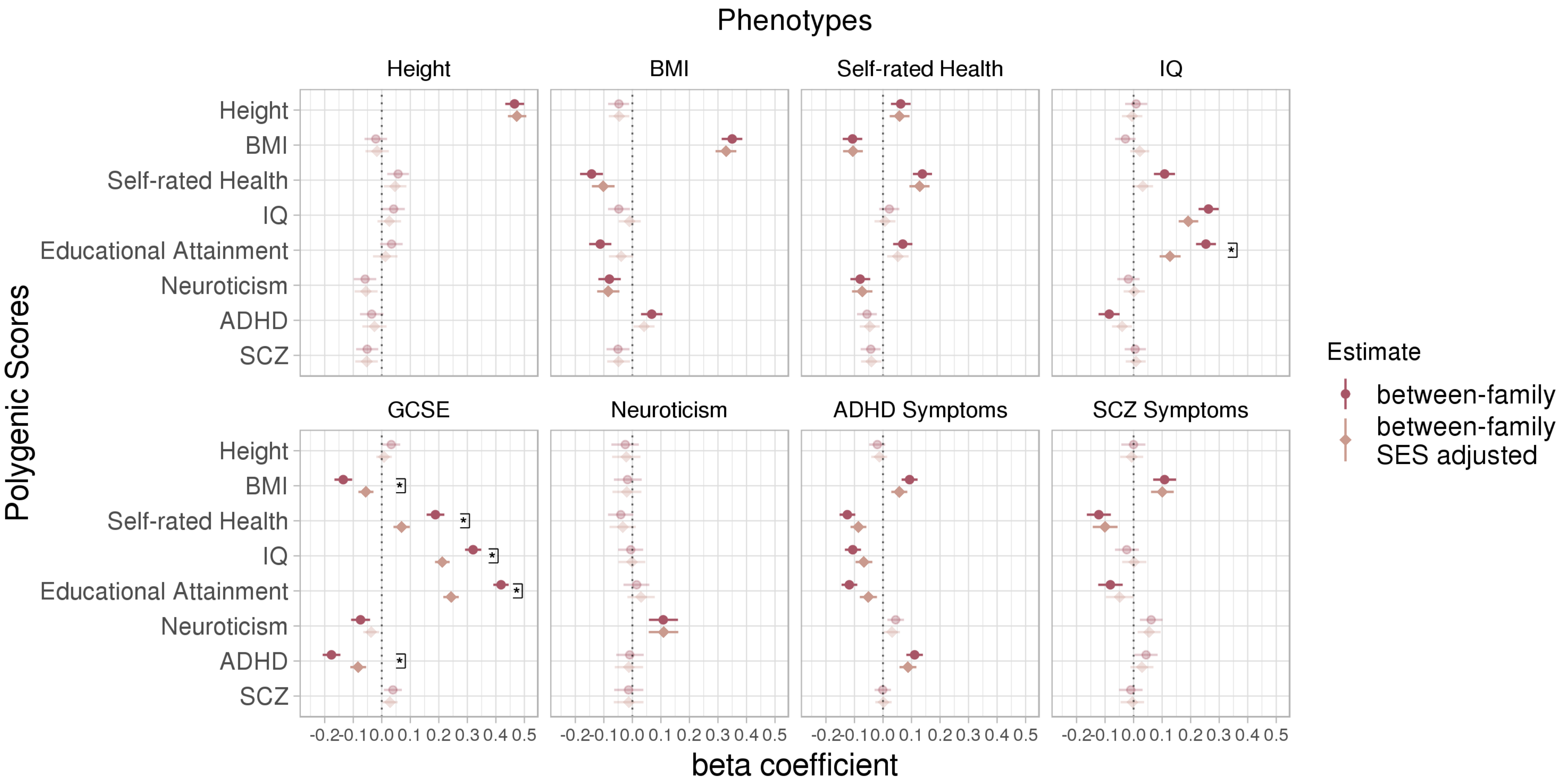

Note. Between-family prediction estimates of eight developmental outcomes using eight polygenic scores before and after statistical correction for family socio-economic status. Genome-wide Polygenic Scores are presented on the y-axis, predicting each of the eight phenotypic traits. Error bars are 95% bootstrap percentile intervals based on 10,000 bootstrap samples (random resampling of DZ twin pairs with replacement). Opaque estimates indicate statistical significance at the false discovery rate corrected threshold of $p < 0.01$. Brackets indicate a significant difference between the two between-family prediction estimates, and only significant differences are shown where at least one of the estimates is significant at the false discovery rate corrected threshold of $p < 0.01$. The dotted line represents a beta coefficient of zero. BMI = Body Mass Index; IQ = Intelligence; GCSE = General Certificate of Secondary Education (educational achievement); ADHD = Attention-Deficit/Hyperactivity Disorder; SCZ = Schizophrenia.

Figure S3. Within- and between-family prediction estimates accounting for separate measures of family socio-economic status

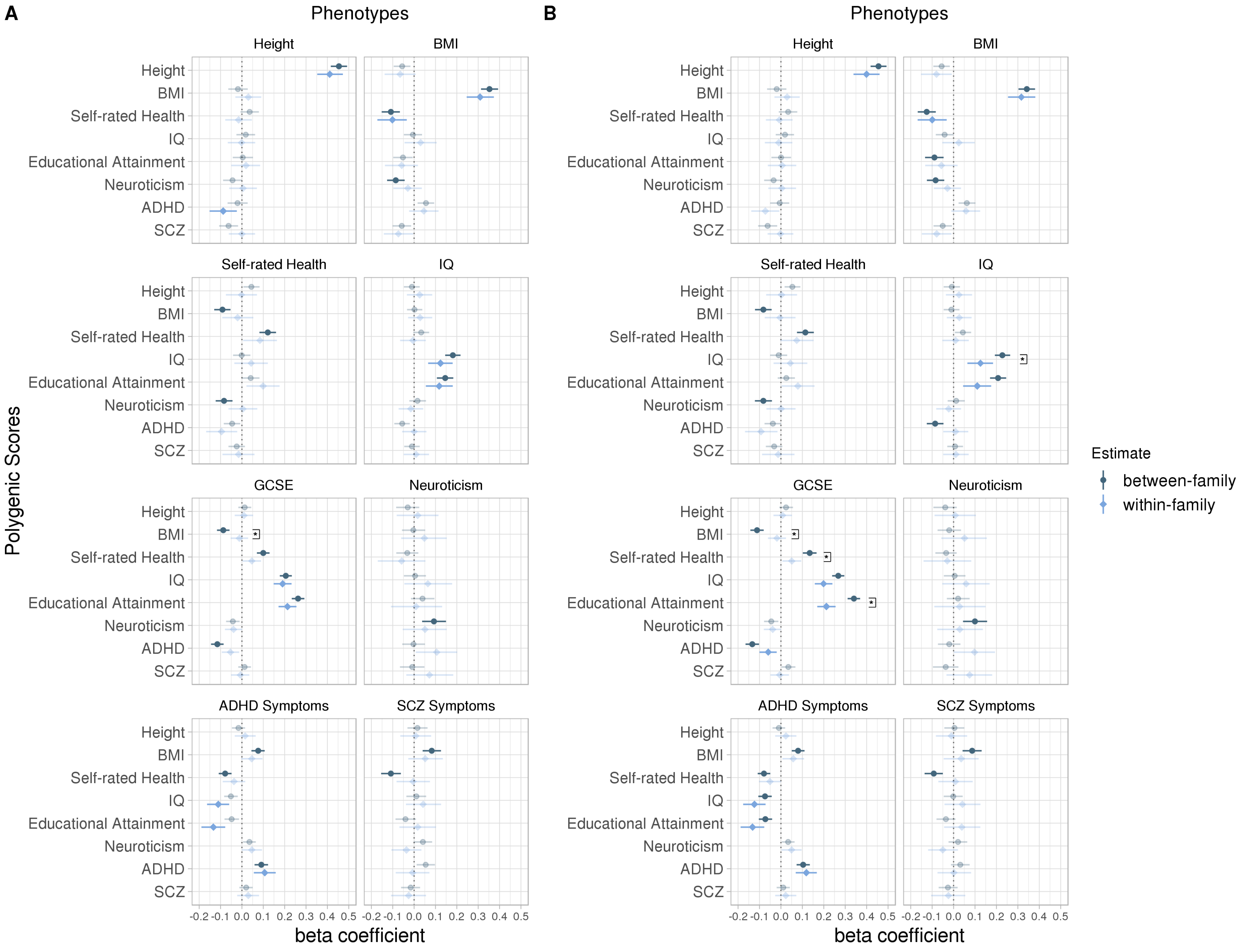

Note. Within- and between-family prediction estimates of eight developmental outcomes using eight polygenic scores after statistically correcting for (A) parental education and (B) parental occupation. Genome-wide Polygenic Scores are presented on the y-axis, predicting each of the eight phenotypic traits. Error bars are 95% bootstrap percentile intervals based on 10,000 bootstrap samples (random resampling of DZ twin pairs with replacement). Opaque estimates indicate statistical significance at the false discovery rate corrected threshold of $p < 0.01$. Brackets indicate a significant difference between within- and between-family prediction estimate, and only significant differences are shown where at least one of the estimates is significant at the false discovery rate corrected threshold of $p < 0.01$. The dotted line represents a beta coefficient of zero. BMI = Body Mass Index; IQ = Intelligence; GCSE = General Certificate of Secondary Education (educational achievement); ADHD = Attention-Deficit/Hyperactivity Disorder; SCZ = Schizophrenia

Figure S4. Within- and between-family prediction estimates for same-sex twin pairs

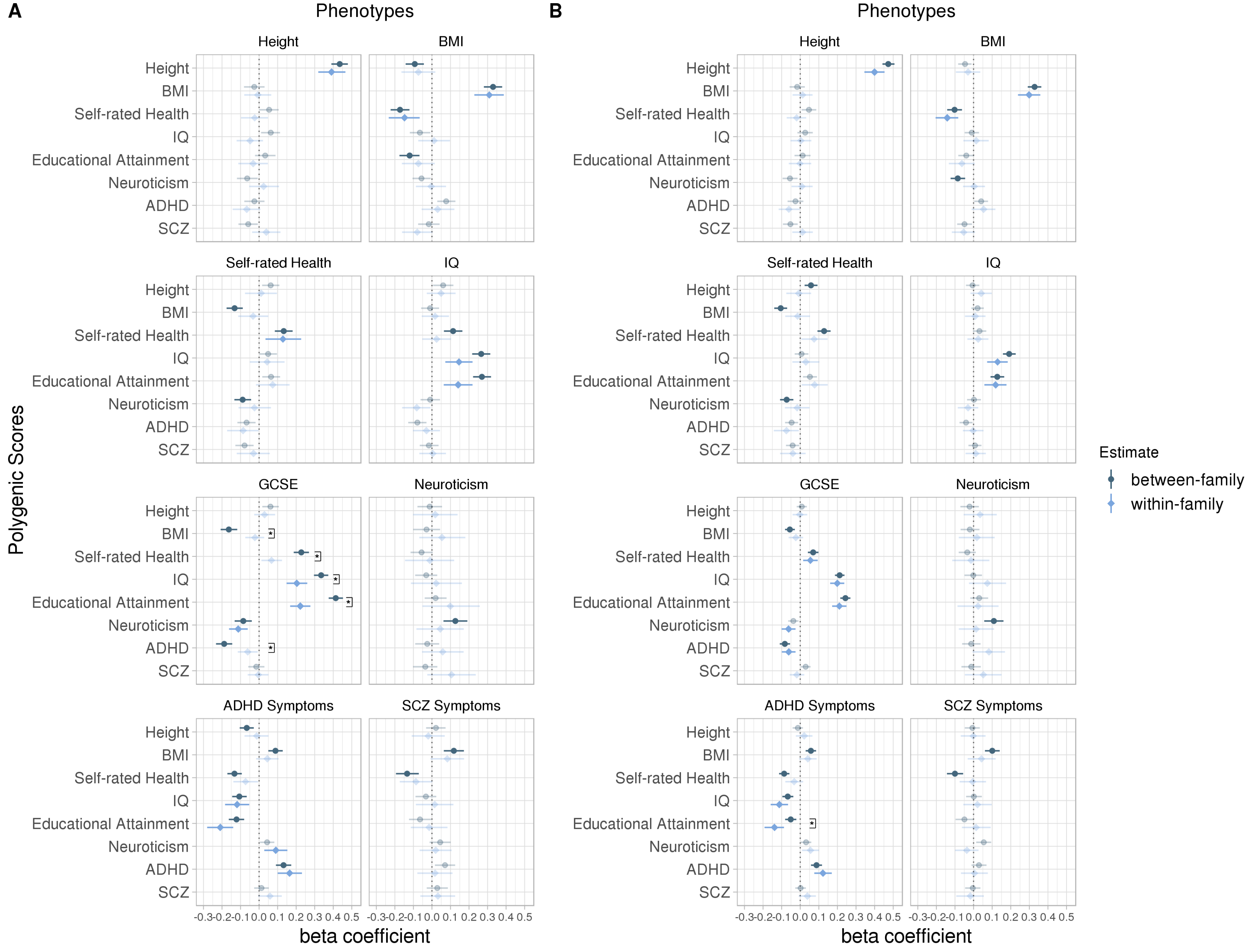

Note. Within- and between-family prediction estimates of eight developmental outcomes using eight polygenic scores before (A) and after (B) statistical correction for family socio-economic status, based on same-sex twin pairs only. Genome-wide Polygenic Scores are presented on the y-axis, predicting each of the eight phenotypic traits. Error bars are 95% bootstrap percentile intervals based on 10,000 bootstrap samples (random resampling of DZ twin pairs with replacement). Opaque estimates indicate statistical significance at the false discovery rate corrected threshold of $p < 0.01$. Brackets indicate a significant difference between within- and between-family prediction estimate, and only significant differences are shown where at least one of the estimates is significant at the false discovery rate corrected threshold of $p < 0.01$. The dotted line represents a beta coefficient of zero. BMI = Body Mass Index; IQ = Intelligence; GCSE = General Certificate of Secondary Education (educational achievement); ADHD = Attention-Deficit/Hyperactivity Disorder; SCZ = Schizophrenia.

Figure S5. Within- and between-family prediction estimates for opposite-sex twin pairs

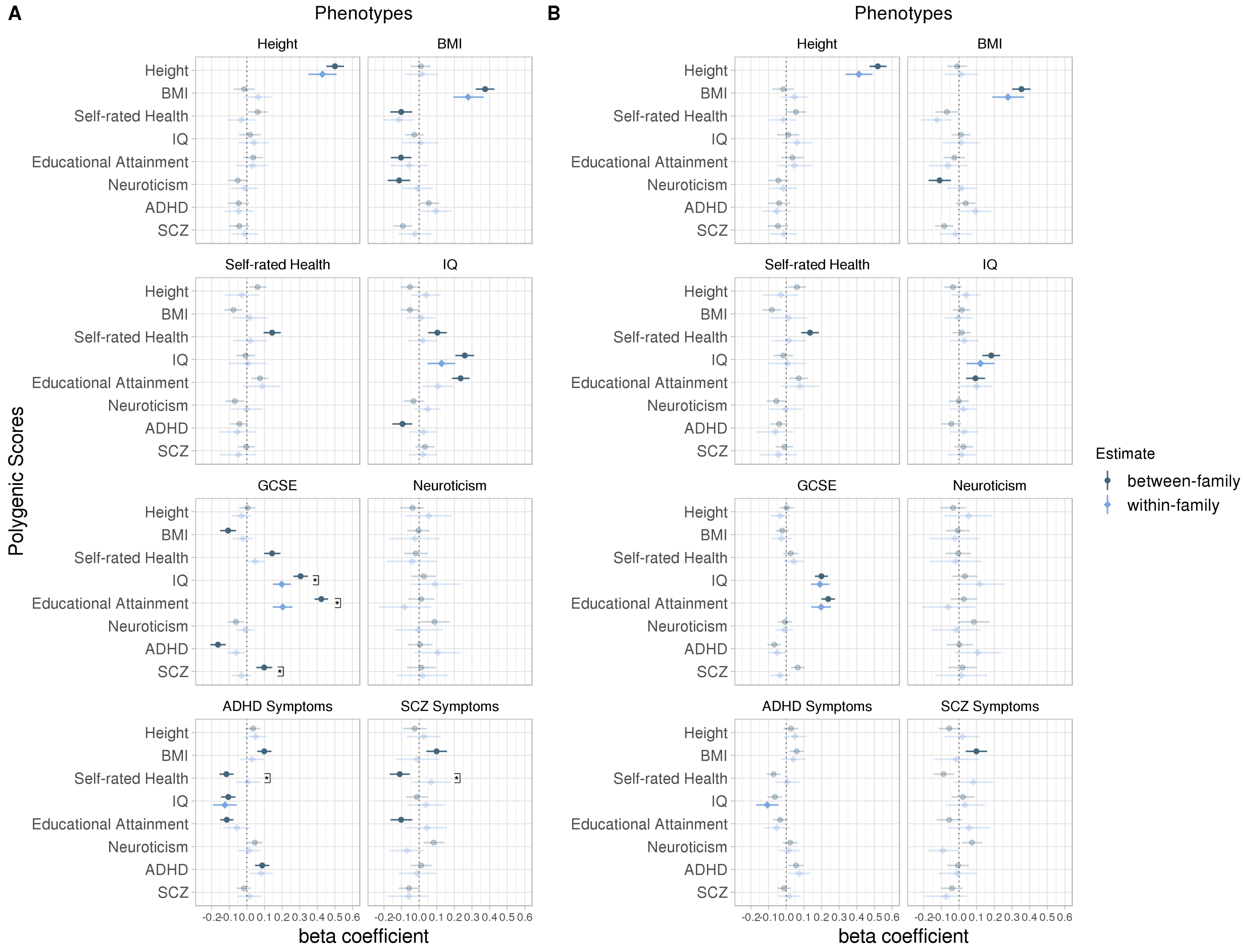

Note. Within- and between-family prediction estimates of eight developmental outcomes using eight polygenic scores before (A) and after (B) statistical correction for family socio-economic status, based on opposite-sex twin pairs only. Genome-wide Polygenic Scores are presented on the y-axis, predicting each of the eight phenotypic traits. Error bars are 95% bootstrap percentile intervals based on 10,000 bootstrap samples (random resampling of DZ twin pairs with replacement). Opaque estimates indicate statistical significance at the false discovery rate corrected threshold of $p < 0.01$. Brackets indicate a significant difference between within- and between-family prediction estimate, and only significant differences are shown where at least one of the estimates is significant at the false discovery rate corrected threshold of $p < 0.01$. The dotted line represents a beta coefficient of zero. BMI = Body Mass Index; IQ = Intelligence; GCSE = General Certificate of Secondary Education (educational achievement); ADHD = Attention-Deficit/Hyperactivity Disorder; SCZ = Schizophrenia.

Figure S6. Within- and between-family prediction estimates based on twin pairs genotyped on OEE chip

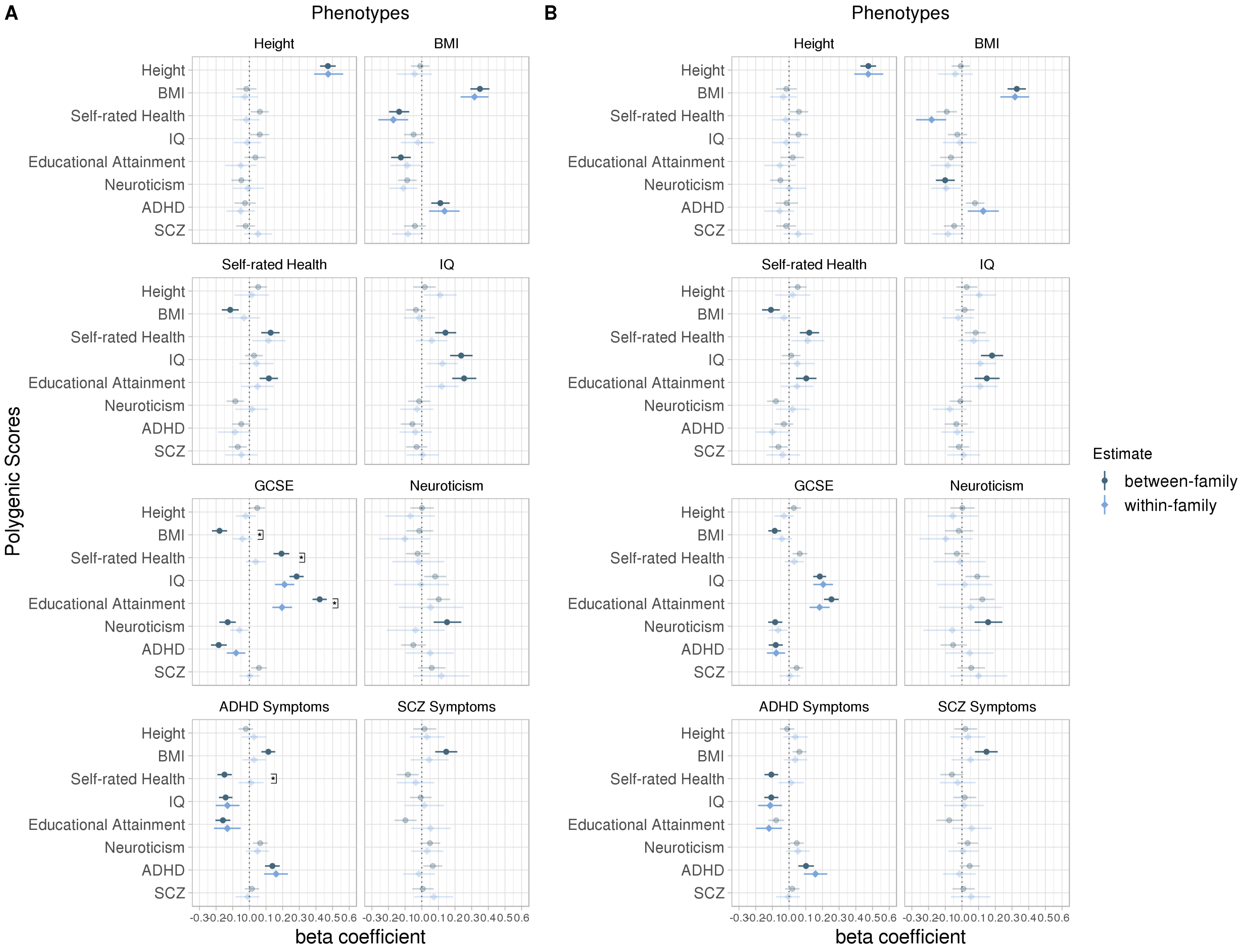

Note. Within- and between-family prediction estimates of eight developmental outcomes using eight polygenic scores before (A) and after (B) statistical correction for family socio-economic status, based on twin pairs genotyped on OEE chip. Genome-wide Polygenic Scores are presented on the y-axis, predicting each of the eight phenotypic traits. Error bars are 95% bootstrap percentile intervals based on 10,000 bootstrap samples (random resampling of DZ twin pairs with replacement). Opaque estimates indicate statistical significance at the false discovery rate corrected threshold of $p < 0.01$. Brackets indicate a significant difference between within- and between-family prediction estimate, and only significant differences are shown where at least one of the estimates is significant at the false discovery rate corrected threshold of $p < 0.01$. The dotted line represents a beta coefficient of zero. BMI = Body Mass Index; IQ = Intelligence; GCSE = General Certificate of Secondary Education (educational achievement); ADHD = Attention-Deficit/Hyperactivity Disorder; SCZ = Schizophrenia.

Figure S7. Within- and between-family prediction estimates based on twin pairs genotyped on OEE chip and the co-twin genotyped on the Affymetrix chip

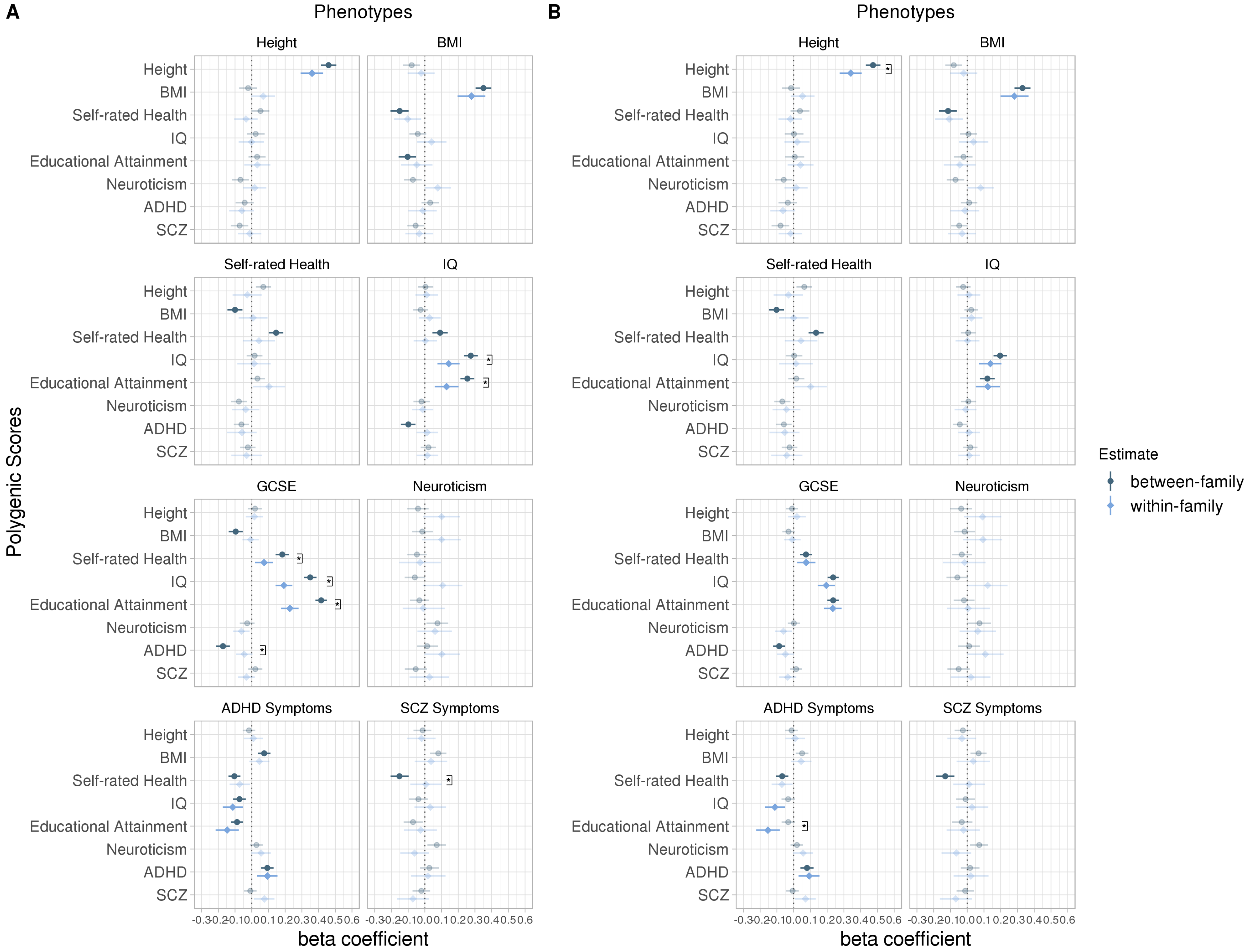

Note. Within- and between-family prediction estimates of eight developmental outcomes using eight polygenic scores before (A) and after (B) statistical correction for family socio-economic status, based on twin pairs with one twin genotyped on OEE chip and the other twin genotyped on Affymetrix chip. Genome-wide Polygenic Scores are presented on the y-axis, predicting each of the eight phenotypic traits. Error bars are 95% bootstrap percentile intervals based on 10,000 bootstrap samples (random resampling of DZ twin pairs with replacement). Opaque estimates indicate statistical significance at the false discovery rate corrected threshold of $p < 0.01$. Brackets indicate a significant difference between within- and between-family prediction estimate, and only significant differences are shown where at least one of the estimates is significant at the false discovery rate corrected threshold of $p < 0.01$. The dotted line represents a beta coefficient of zero. BMI = Body Mass Index; IQ = Intelligence; GCSE = General Certificate of Secondary Education (educational achievement); ADHD = Attention-Deficit/Hyperactivity Disorder; SCZ = Schizophrenia

Figure S8. Within- and between-family coefficients estimated using a SNP-kinship matrix as random effect

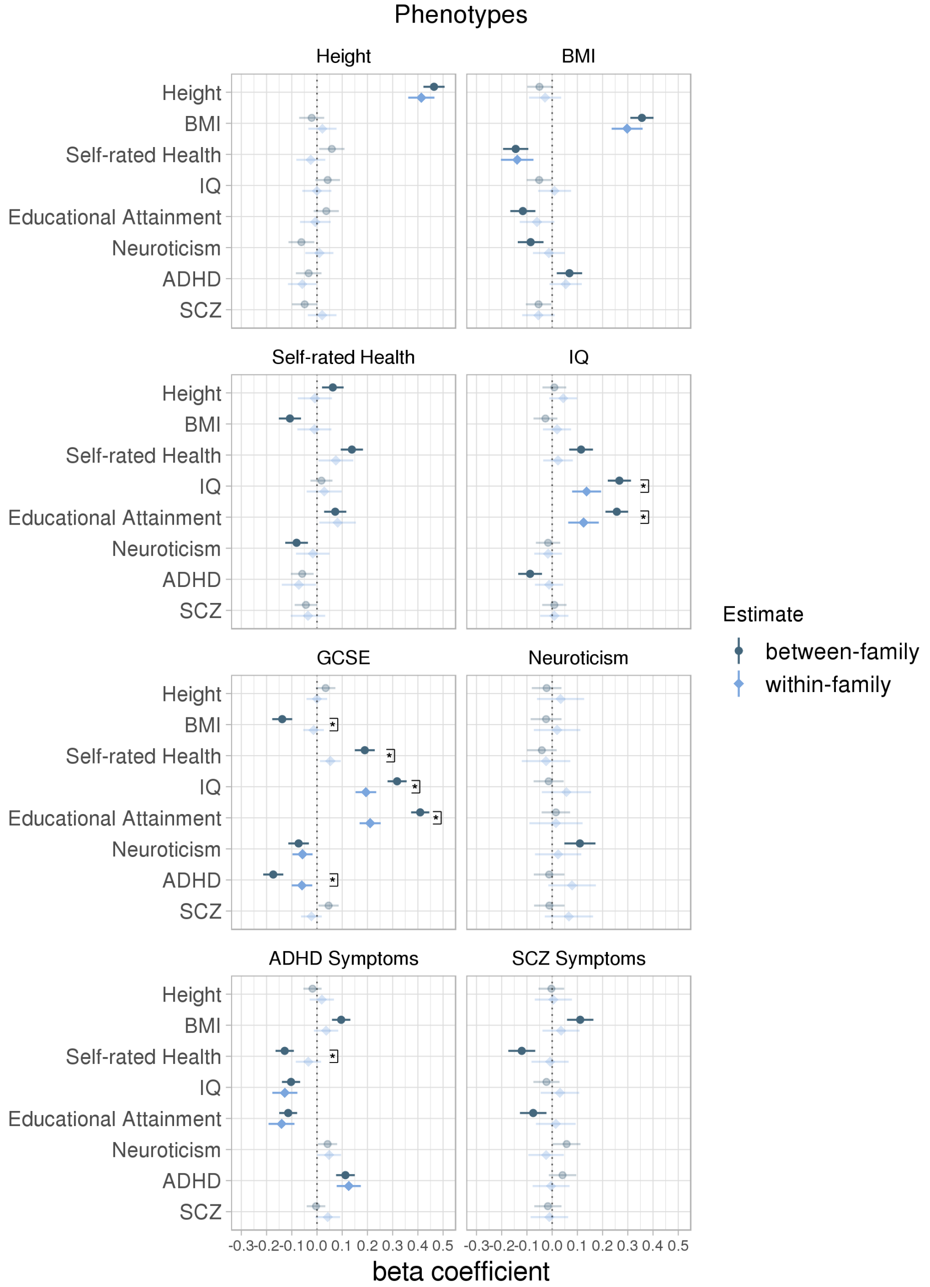

Note. Within- and between-family prediction estimates of eight developmental outcomes using eight polygenic scores. Genome-wide Polygenic Scores are presented on the y-axis, predicting each of the eight phenotypic traits. Error bars are 95% confidence intervals calculated using the standard errors of the fixed effects. Opaque estimates indicate statistical significance at the false discovery rate corrected threshold of $p < 0.01$. Brackets indicate a significant difference between within- and between-family prediction estimate, and only significant differences are shown where at least one of the estimates is significant at the false discovery rate corrected threshold of $p < 0.01$. The dotted line represents a beta coefficient of zero. BMI = Body Mass Index; IQ = Intelligence; GCSE = General Certificate of Secondary Education (educational achievement); ADHD = Attention-Deficit/Hyperactivity Disorder; SCZ = Schizophrenia.

Figure S9. Within- and between-family prediction estimates using polygenic scores calculated with causal fraction of markers of 0.1

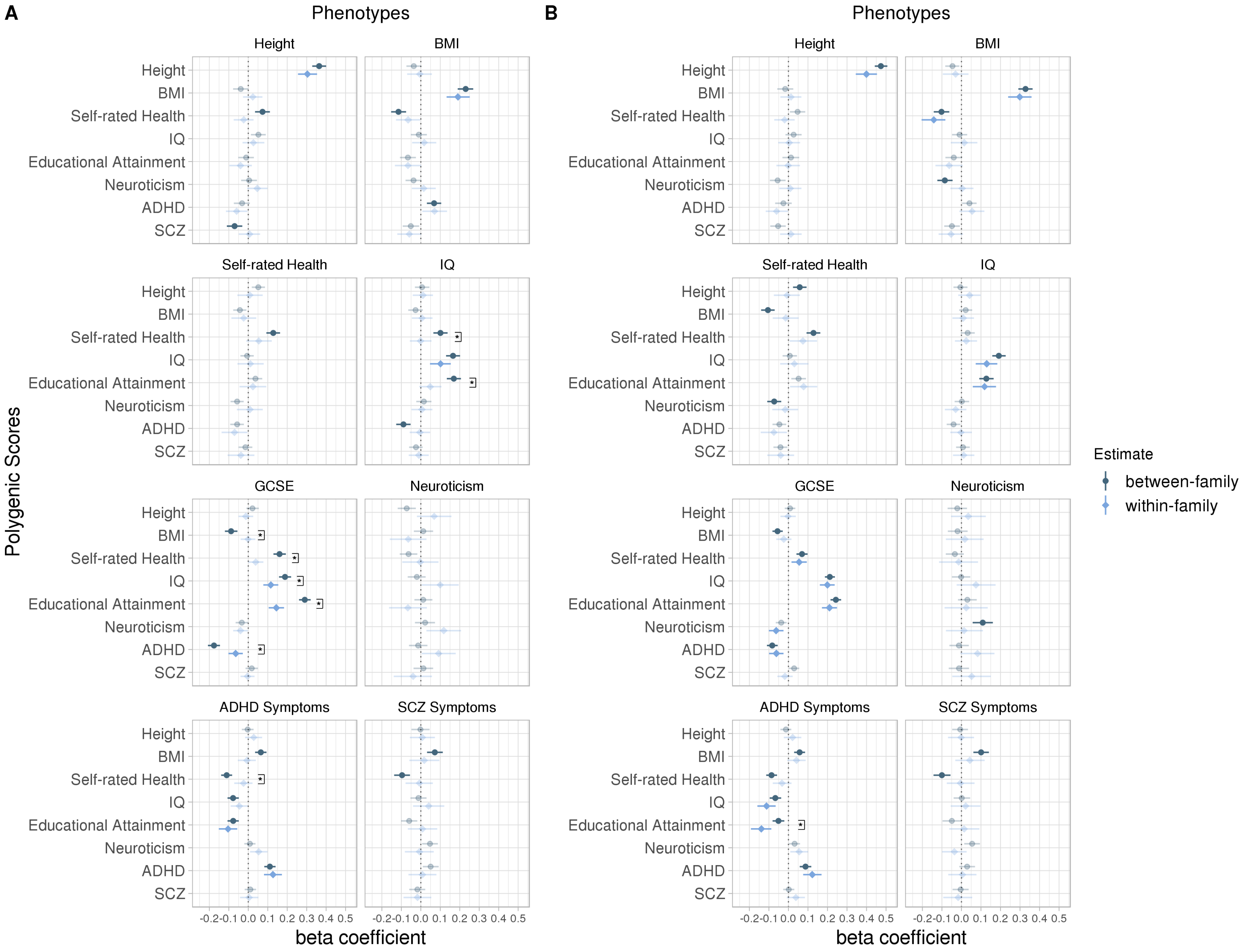

Note. Within- and between-family prediction estimates of eight developmental outcomes using eight polygenic scores before (A) and after (B) statistical correction for family socio-economic status, using polygenic scores calculated with causal fraction of markers of 0.1. Genome-wide Polygenic Scores are presented on the y-axis, predicting each of the eight phenotypic traits. Error bars are 95% bootstrap percentile intervals based on 10,000 bootstrap samples (random resampling of DZ twin pairs with replacement). Opaque estimates indicate statistical significance at the false discovery rate corrected threshold of $p < 0.01$. Brackets indicate a significant difference between within- and between-family prediction estimate, and only significant differences are shown where at least one of the estimates is significant at the false discovery rate corrected threshold of $p < 0.01$. The dotted line represents a beta coefficient of zero. BMI = Body Mass Index; IQ = Intelligence; GCSE = General Certificate of Secondary Education (educational achievement); ADHD = Attention-Deficit/Hyperactivity Disorder; SCZ = Schizophrenia.

Figure S10. Within- and between-family prediction estimates based on polygenic scores with UK Biobank or all UK discovery samples removed

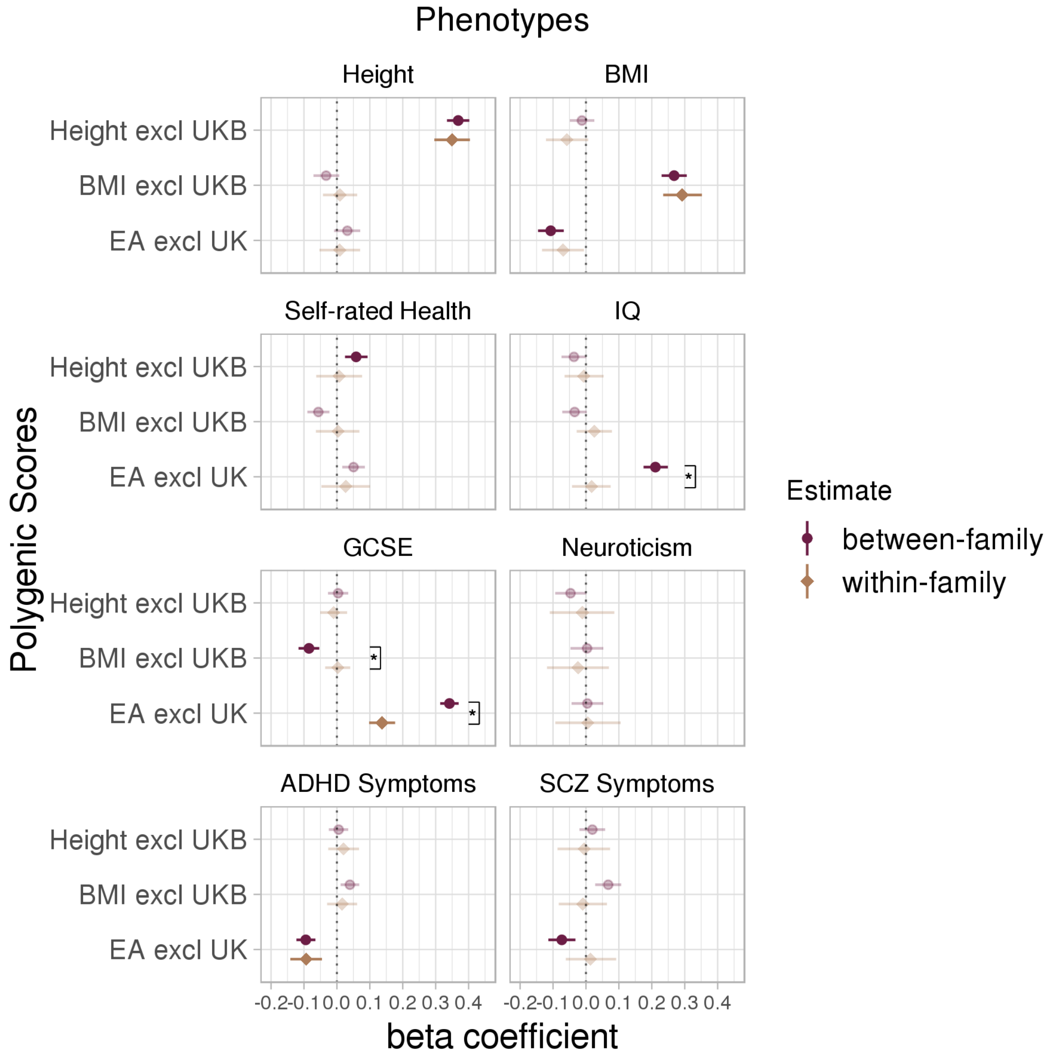

Note. Within- and between-family prediction estimates of eight developmental outcomes in using polygenic scores with UK Biobank or all UK samples removed at discovery stage. Genome-wide Polygenic Scores are presented on the y-axis, predicting each of the eight phenotypic traits. Error bars are 95% bootstrap percentile intervals based on 10,000 bootstrap samples (random resampling of DZ twin pairs with replacement). Opaque estimates indicate statistical significance at the false discovery rate corrected threshold of $p < 0.01$. Brackets indicate a significant difference between within- and between-family prediction estimate, and only significant differences are shown where at least one of the estimates is significant at the false discovery rate corrected threshold of $p < 0.01$. The dotted line represents a beta coefficient of zero. BMI = Body Mass Index; IQ = Intelligence; GCSE = General Certificate of Secondary Education (educational achievement); ADHD = Attention-Deficit/Hyperactivity Disorder; SCZ = Schizophrenia; EA = Educational Attainment; UKB = UK Biobank.

Supplementary Tables

Table S1. Descriptive statistics, age and sex effects for phenotypes

	N Pairs	Mean	SD	Skew	Min	Max	F sex	P sex	R^2 sex	F age	P age	R^2 age	P order	R^2 order
**Height**	1,463	171.997	10.457	0.142	132	211	1,460.639	< 0.001	0.500	0.855	0.355	0.001	0.174	<0.001
**BMI**	1,353	23.495	4.676	1.520	12.061	47.477	0.002	0.964	<0.001	16.470	< 0.001	0.012	0.918	<0.001
**Self-rated Health**	1,494	3.480	0.672	-0.330	1.000	5.000	8.188	0.004	0.005	1.457	0.228	0.001	0.987	<0.001
**IQ**	1,569	0.117	0.954	-0.242	-3.441	3.040	7.277	0.007	0.005	104.278	< 0.001	0.062	0.125	<0.001
**GCSE**	2,366	8.952	1.194	-0.330	4.670	11.000	1.814	0.178	<0.001	4.49	0.034	0.001	0.487	<0.001
**Neuroticism**	789	2.583	0.655	0.280	1.000	5.000	31.894	< 0.001	0.039	2.202	0.138	0.003	0.740	<0.001
**ADHD Symptoms**	2,469	0.063	1.002	1.357	-1.371	5.066	159.896	< 0.001	0.061	16.877	< 0.001	0.007	0.073	<0.001
**SCZ Symptoms**	1,140	-0.026	0.705	1.560	-0.816	4.093	2.041	0.153	0.002	6.122	0.013	0.005	0.858	<0.001
**SES**	2,962	0.209	0.994	0.046	-2.351	2.495	--	--	--	--	--	--	--	--

Note. Means and standard deviations for individual measures are calculated based on raw data. Height, BMI, self-reported health, GCSE grades and neuroticism means and standard deviations are reported on their original scale. IQ, ADHD symptoms, schizophrenia symptoms and socioeconomic status are reported on the z-scale as standardization was required to form the composite. Sex, age and birth order tests were performed on one randomly selected twin per pair. R^2= proportion of variance explained. Order = birth order; BMI = Body Mass Index; IQ = Intelligence; GCSE = General Certificate of Secondary Education (educational achievement); ADHD = Attention-Deficit/Hyperactivity Disorder; SCZ = Schizophrenia; SES = family socio-economic status.

Table S2. GWAS used for polygenic score calculation

Trait	Year	SNP-h^2	GPS R^2	Cases	Controls	GWAS sample size	Overlapping SNPs[a]	Notes
BMI[2]	2018	22.4% (3.7%)[2]	10.2%[2]	-	-	681,275	424,816	-
Height[2]	2018	48.3% (3.7%)[2]	24.4%[2]	-	-	693,529	427,030	-
Self-rated health[3]	2018	13% (0. 6%)[4]	--	-	-	337,199	499,422	-
Intelligence[5]	2018	19% (1%)[5]	6.7%[6]	-	-	266,453	497,059	GWAS re-run excluding TEDS sample (3,414)
Educational Attainment[7]	2018	12.2% (0.3%)[7]	11.4% [7]	-	-	766,345	496,633	-
Neuroticism[8]	2017	10.8% (0.5%)[8]	2.8% [8]	-	-	329,821	500,849	-
ADHD[9]	2019	21.6% (1.4%)[9]	5.5%[9]	20,183	35,191	55,374	469,352	-
Schizophrenia[10]	2018	20% (0.6%)[10]	5.7%[10]	40,675	64,643	105,318	487,423	-

Note. H^2 = heritability; R^2 = phenotypic variance explained; [a] = number of overlapping SNPs between GWA studies and TEDS individual-level genotype data.

Table S3. Intraclass coefficients for same-sex and opposite-sex twin pairs

	Same-sex twin pairs				Opposite-sex twin pairs			
**Phenotype**	**N pairs**	**ICC**	**ICC 95% CI L**	**ICC 95% CI U**	**N pairs**	**ICC**	**ICC 95% CI L**	**ICC 95% CI U**
**Height**	789	0.435	0.365	0.518	674	0.443	0.367	0.534
**BMI**	733	0.339	0.271	0.424	620	0.286	0.215	0.381
**Self-rated Health**	805	0.182	0.124	0.266	689	0.083	0.036	0.193
**IQ**	824	0.451	0.381	0.532	745	0.386	0.316	0.47
**GCSE**	1,220	0.579	0.517	0.647	1,146	0.585	0.521	0.656
**Neuroticism**	429	0.084	0.025	0.282	360	0.123	0.054	0.281
**ADHD Symptoms**	1,285	0.328	0.275	0.391	1,184	0.317	0.262	0.382
**SCZ Symptoms**	613	0.308	0.235	0.403	527	0.194	0.124	0.302

Note. BMI = Body Mass Index; IQ = Intelligence; GCSE = General Certificate of Secondary Education (educational achievement); ADHD = Attention-Deficit/Hyperactivity Disorder; SCZ = Schizophrenia symptoms; EA = Educational Attainment; Neurot = Neuroticism; ICC = Intraclass coefficient; CI = Confidence Interval.

Table S4. Intraclass coefficients for twin pairs split by genotyping chip

	Twin pairs with both twins genotyped on OEE				Twin pairs with one twin genotyped on OEE and the other twin genotyped on Affy			
**Phenotype**	**N pairs**	**ICC**	**ICC 95% CI L**	**ICC 95% CI U**	**N pairs**	**ICC**	**ICC 95% CI L**	**ICC 95% CI U**
**Height**	650	0.401	0.326	0.492	813	0.468	0.398	0.549
**BMI**	603	0.331	0.257	0.426	750	0.297	0.232	0.381
**Self-rated Health**	665	0.172	0.111	0.267	829	0.107	0.056	0.201
**IQ**	510	0.391	0.308	0.496	1059	0.437	0.376	0.508
**GCSE**	1050	0.57	0.504	0.644	1316	0.59	0.531	0.656
**Neuroticism**	304	0	0	0	485	0.167	0.098	0.284
**ADHD Symptoms**	1047	0.312	0.255	0.382	1422	0.329	0.278	0.388
**SCZ Symptoms**	490	0.241	0.165	0.35	650	0.263	0.195	0.356

Note. BMI = Body Mass Index; IQ = Intelligence; GCSE = General Certificate of Secondary Education (educational achievement); ADHD = Attention-Deficit/Hyperactivity Disorder; SCZ = Schizophrenia symptoms; EA = Educational Attainment; Neurot = Neuroticism; ICC = Intraclass coefficient; CI = Confidence Interval; OEE = Illumina HumanOmniExpressExome-8v1.2 chip; Affy = AffymetrixGeneChip 6.0 SNP chip.

Table S5. Intraclass coefficients

Phenotype	N pairs	ICC	ICC 95% CI L	ICC 95% CI U
**Height**	1,463	0.439	0.386	0.498
**BMI**	1,353	0.317	0.265	0.379
**Self-rated Health**	1,494	0.136	0.093	0.199
**IQ**	1,569	0.422	0.371	0.479
**GCSE**	2,366	0.582	0.537	0.63
**Neuroticism**	789	0.103	0.055	0.193
**ADHD Symptoms**	2,469	0.323	0.285	0.366
**SCZ Symptoms**	1,140	0.254	0.201	0.32

Note. BMI = Body Mass Index; IQ = Intelligence; GCSE = General Certificate of Secondary Education (educational achievement); ADHD = Attention-Deficit/Hyperactivity Disorder; SCZ = Schizophrenia symptoms; ICC = Intraclass coefficient; CI = Confidence Interval.

Table S12. Within- and between-family prediction estimates of family socio-economic status

pheno	GPS	beta.B	L.CI.B	U.CI.B	P.B	beta.W	L.CI.W	U.CI.W	P.W	P.Diff
SES	ADHD	-0.168	-0.214	-0.13	1.32e-28	0	0	0	1	2.69e-08
SES	BMI	-0.19	-0.216	-0.134	3.80e-38	0	0	0	1	1.80e-10
SES	EA	0.433	0.409	0.479	2.35e-204	0	0	0	1	5.47e-49
SES	Height	0.069	0.02	0.102	6.51e-06	0	0	0	1	0.025
SES	IQ	0.231	0.213	0.291	4.28e-55	0	0	0	1	7.65e-14
SES	Neurot	-0.06	-0.129	-0.043	5.35e-05	0	0	0	1	0.049
SES	SCZ	0.04	-0.015	0.066	0.008	0	0	0	1	0.201
SES	SRH	0.302	0.238	0.318	3.86e-89	0	0	0	1	2.24e-23

Note. SES = family socio-economic status; BMI = Body Mass Index; IQ = Intelligence; GCSE = General Certificate of Secondary Education (educational achievement); ADHD = Attention-Deficit/Hyperactivity Disorder; SCZ = Schizophrenia symptoms; EA = Educational Attainment; Neurot = Neuroticism; SRH = Self-rated Health; B = Between-family estimate; W = Within-family estimate; L.CI = Lower 95% bootstrap percentile interval; U.CI = Upper 95% bootstrap percentile interval; P = statistical significance of beta estimate; P.diff = statistical significance of difference between within- and between-family estimates. Fixed effects are estimated using multiple regression analysis as parental SES is invariant within DZ twin pairs and thus mixed-effects modelling is not possible.

Table S24. Within- and between-family prediction estimates based on polygenic scores with UK Biobank or all UK discovery samples removed

pheno	GPS	beta.B	L.CI.B	U.CI.B	P.B	beta.W	L.CI.W	U.CI.W	P.W	TotEff	PercRed	BetaDiff	Diff.L.CI	Diff.U.CI	P.diff
ADHD	BMI excl UKB	0.039	0.011	0.068	3.64e-02	0.016	-0.03	0.061	5.04e-01	0.023	60.52	0.024	-0.029	0.076	4.26e-01
ADHD	EA excl all UK	-0.095	-0.123	-0.065	2.93e-07	-0.093	-0.141	-0.045	2.44e-04	-0.094	1.15	-0.001	-0.057	0.056	9.72e-01
ADHD	Height excl UKB	0.005	-0.024	0.034	7.73e-01	0.02	-0.026	0.067	4.14e-01	0.015	-274.51	-0.015	-0.07	0.041	6.33e-01
BMI	BMI excl UKB	0.268	0.23	0.306	2.71e-26	0.292	0.234	0.352	2.18e-21	0.285	-9.07	-0.024	-0.096	0.047	5.34e-01
BMI	EA excl all UK	-0.107	-0.145	-0.067	2.15e-05	-0.069	-0.133	-0.006	4.56e-02	-0.081	35.51	-0.038	-0.111	0.037	3.73e-01
BMI	Height excl UKB	-0.012	-0.049	0.025	6.18e-01	-0.058	-0.121	0.007	8.12e-02	-0.043	-370.21	0.046	-0.028	0.118	2.70e-01
GCSE	BMI excl UKB	-0.085	-0.117	-0.053	7.53e-05	0.003	-0.036	0.04	8.87e-01	-0.048	103.15	-0.088	-0.136	-0.039	2.08e-03
GCSE	EA excl all UK	0.342	0.314	0.37	3.23e-64	0.137	0.098	0.177	2.15e-11	0.249	59.88	0.205	0.155	0.254	4.46e-13
GCSE	Height excl UKB	0.004	-0.027	0.034	8.58e-01	-0.01	-0.051	0.031	6.16e-01	-0.002	369.05	0.014	-0.037	0.064	6.33e-01
SRH	BMI excl UKB	-0.056	-0.09	-0.023	1.27e-02	0.004	-0.064	0.068	9.06e-01	-0.004	106.98	-0.06	-0.132	0.016	1.34e-01
SRH	EA excl all UK	0.051	0.016	0.085	2.51e-02	0.027	-0.047	0.1	4.66e-01	0.03	46.46	0.023	-0.057	0.105	5.88e-01
SRH	Height excl UKB	0.058	0.025	0.093	7.49e-03	0.007	-0.063	0.076	8.48e-01	0.014	88.27	0.052	-0.025	0.129	2.18e-01
Height	BMI excl UKB	-0.033	-0.071	0.007	2.04e-01	0.009	-0.042	0.062	7.39e-01	-0.009	127.78	-0.042	-0.108	0.024	2.65e-01
Height	EA excl all UK	0.032	-0.009	0.071	2.16e-01	0.009	-0.053	0.07	7.77e-01	0.019	73.04	0.023	-0.05	0.095	5.59e-01
Height	Height excl UKB	0.368	0.334	0.402	6.29e-54	0.35	0.296	0.404	4.03e-35	0.358	5.02	0.019	-0.047	0.083	6.05e-01
IQ	BMI excl UKB	-0.034	-0.072	0.002	1.65e-01	0.025	-0.028	0.079	3.44e-01	0	173.67	-0.06	-0.125	0.007	1.01e-01
IQ	EA excl all UK	0.211	0.175	0.249	1.27e-18	0.017	-0.042	0.075	5.63e-01	0.095	91.83	0.194	0.126	0.265	3.41e-07
IQ	Height excl UKB	-0.037	-0.074	0.001	1.33e-01	-0.007	-0.065	0.054	8.21e-01	-0.019	82.19	-0.03	-0.1	0.038	4.25e-01
Neurot	BMI excl UKB	0.003	-0.047	0.053	9.15e-01	-0.024	-0.118	0.07	6.11e-01	-0.021	825.01	0.027	-0.08	0.134	6.27e-01
Neurot	EA excl all UK	0.004	-0.044	0.053	8.88e-01	0.005	-0.093	0.105	9.17e-01	0.005	-25.51	-0.001	-0.112	0.109	9.85e-01
Neurot	Height excl UKB	-0.047	-0.093	0.001	1.12e-01	-0.011	-0.11	0.086	8.27e-01	-0.015	76.63	-0.036	-0.143	0.073	5.36e-01
SCZ	BMI excl UKB	0.068	0.028	0.107	1.40e-02	-0.01	-0.083	0.064	7.88e-01	0.01	114.1	0.077	-0.01	0.163	8.55e-02

SCZ	EA excl all UK	-0.073	-0.114	-0.032	6.90e-03	0.014	-0.061	0.092	7.31e-01	-0.008	118.91	-0.087	-0.178	0.002	7.22e-02
SCZ	Height excl UKB	0.019	-0.02	0.058	4.57e-01	-0.005	-0.086	0.073	8.92e-01	0.001	126.82	0.025	-0.062	0.112	5.96e-01

Note. BMI = Body Mass Index; IQ = Intelligence; GCSE = General Certificate of Secondary Education (educational achievement); ADHD = Attention-Deficit/Hyperactivity Disorder; SCZ = Schizophrenia symptoms; EA = Educational Attainment; Neurot = Neuroticism; SRH = Self-rated Health; UKB = UK Biobank; B = Between-family estimate; W = Within-family estimate; L.CI = Lower 95% bootstrap percentile interval; U.CI = Upper 95% bootstrap percentile interval; P = statistical significance of beta estimate; TotEff = Total effect derived as the intra-class correlation weighted sum of the within- and between family effect. PercRed = Reduction of prediction estimates when comparing within- to between-family estimates in percentage. BetaDiff = Difference of between-family and within-family beta coefficient; P.diff = statistical significance of difference between within- and between-family estimates.

Table S25 Phenotypic and polygenic score mean differences by polygenic score difference quantiles

phenotype	GPS quant	mean phen	CI.L	CI.U	mean GPS	CI.L	CI.U
Height	1	0.270	-1.339	1.880	-0.004	-0.016	0.008
Height	2	1.718	0.203	3.233	-0.003	-0.033	0.027
Height	3	2.435	0.738	4.132	0.020	-0.030	0.071
Height	4	3.489	2.039	4.939	-0.030	-0.102	0.041
Height	5	1.433	-0.209	3.075	0.066	-0.025	0.158
Height	6	1.452	-0.144	3.048	0.019	-0.101	0.139
Height	7	4.620	2.862	6.379	-0.007	-0.154	0.141
Height	8	3.649	1.813	5.485	-0.038	-0.218	0.142
Height	9	7.021	5.316	8.727	0.065	-0.165	0.295
Height	10	8.989	7.181	10.798	-0.090	-0.422	0.242
BMI	1	0.064	-0.843	0.972	0.001	-0.012	0.014
BMI	2	0.968	0.126	1.810	0.004	-0.029	0.037
BMI	3	-0.555	-1.439	0.329	-0.011	-0.066	0.044
BMI	4	1.204	0.319	2.089	-0.027	-0.105	0.051
BMI	5	0.689	-0.073	1.451	0.055	-0.047	0.158
BMI	6	0.885	-0.009	1.778	-0.031	-0.159	0.097
BMI	7	1.495	0.584	2.406	0.075	-0.084	0.235
BMI	8	1.309	0.410	2.208	-0.084	-0.281	0.113
BMI	9	1.762	0.999	2.526	0.043	-0.200	0.286
BMI	10	2.933	2.092	3.773	-0.026	-0.378	0.327
IQ	1	1.369	-1.040	3.778	0.005	-0.006	0.016
IQ	2	-0.260	-2.860	2.341	-0.011	-0.042	0.019
IQ	3	1.139	-1.385	3.662	0.010	-0.041	0.061
IQ	4	-0.725	-3.287	1.838	-0.009	-0.081	0.063
IQ	5	2.081	-0.367	4.529	0.023	-0.069	0.115
IQ	6	1.483	-0.982	3.949	0.019	-0.099	0.137
IQ	7	1.288	-1.222	3.799	-0.051	-0.193	0.092
IQ	8	2.379	-0.215	4.973	0.092	-0.083	0.268
IQ	9	4.304	1.881	6.728	-0.101	-0.324	0.121
IQ	10	3.291	0.934	5.647	0.023	-0.293	0.340
GCSE	1	0.068	-0.067	0.203	0.005	-0.004	0.013
GCSE	2	-0.057	-0.204	0.090	0.001	-0.023	0.024
GCSE	3	0.195	0.053	0.336	-0.012	-0.051	0.026
GCSE	4	0.080	-0.063	0.223	0.021	-0.033	0.074
GCSE	5	0.097	-0.039	0.234	-0.022	-0.094	0.049
GCSE	6	0.266	0.129	0.403	0.054	-0.039	0.146

GCSE	7	0.226	0.083	0.369	0.084	-0.030	0.199
GCSE	8	0.307	0.180	0.435	0.060	-0.079	0.200
GCSE	9	0.341	0.192	0.490	0.013	-0.159	0.185
GCSE	10	0.471	0.327	0.614	-0.202	-0.445	0.041

Note. BMI = Body Mass Index; IQ = Intelligence; GCSE = General Certificate of Secondary Education (educational achievement); GPS = genome wide polygenic score; quant = quantile; phen = phenotype; CI.L = 95% lower confidence interval; CI.U = 95% upper confidence interval; GPS quant 1 = lowest absolute GPS twin pair difference quantile; GPS quant 10 = highest absolute GPS twin pair difference quantile.

Supplementary Methods

Methods S1. Polygenic score calculation

To calculate polygenic scores, we used a Bayesian approach to polygenic score calculation, implemented in the software *LDpred*[1]. In comparison with conventional clumping and p value thresholding approaches, LDpred has demonstrated an improvement in predictive accuracy[1]. Through this method, a posterior effect size is calculated for each single SNP that is present in both the GWA study summary statistics and the target genotype sample (see Table S2 for number of overlapping SNPs). To calculate the posterior effect size, the original summary statistic effect size estimates are adjusted based on two factors: (a) the relative influence of a SNP given its level of LD with surrounding SNPs in the target sample (here TEDS), and (b) a prior on the effect size of each SNP. To account for LD, we set the radius to a 2 megabase window. The effect size prior depends on the SNP-heritability of the discovery (i.e., GWA study) trait and an assumption on the fraction of causal markers believed to influence the discovery trait. Using the prior, the beta effect sizes are reweighted such that the effects are spread out among the SNPs across the whole genome in proportion to the LD present among these SNPs. To accommodate the high computational demands of these calculations, we reduced our genotype data set to SNPs that had perfect imputation scores (info = 1), leaving 515,100 SNPs for analysis. In the next step, all trait-associated alleles were counted (0, 1, or 2 for each SNP), weighted by the posterior SNP effect size obtained through LDpred, and summed across the genome to calculate a GPS for each individual in TEDS.

References

1. Vilhjalmsson, B.J., Yang, J., Finucane, H.K., Gusev, A., Lindstrom, S., Ripke, S., Genovese, G., Loh, P.-R., Bhatia, G., Do, R., et al. (2015). Modeling Linkage Disequilibrium Increases Accuracy of Polygenic Risk Scores. Am. J. Hum. Genet. 97, 576–592.

2. Yengo, L., Sidorenko, J., Kemper, K.E., Zheng, Z., Wood, A.R., Weedon, M.N., Frayling, T.M., Hirschhorn, J., Yang, J., Visscher, P.M., et al. (2018). Meta-analysis of genome-wide association studies for height and body mass index in similar to 700 000 individuals of European ancestry. Hum. Mol. Genet. 27, 3641–3649.

3. McInnes, G., Tanigawa, Y., DeBoever, C., Lavertu, A., Olivieri, J.E., Aguirre, M., and Rivas, M.A. (2018). Global Biobank Engine: enabling genotype-phenotype browsing for biobank summary statistics. Bioinformatics 9, 1612.

4. Harris, S.E., Hagenaars, S.P., Davies, G., Hill, W.D., Liewald, D.C.M., Ritchie, S.J., Marioni, R.E., Sudlow, C.L.M., Wardlaw, J.M., McIntosh, A.M., et al. (2017). Molecular genetic contributions to self-rated health. Int J Epidemiol 46, 994–1009.

5. Savage, J.E., Jansen, P.R., Stringer, S., Watanabe, K., Bryois, J., de Leeuw, C.A., Nagel, M., Awasthi, S., Barr, P.B., Coleman, J.R.I., et al. (2018). Genome-wide association meta-analysis in 269,867 individuals identifies new genetic and functional links to intelligence. Nat. Genet. 50, 912–919.

6. Allegrini, A., Selzam, S., Rimfeld, K., Stumm, von, S., Pingault, J.-B., and Plomin, R. (2019). Genomic prediction of cognitive traits in childhood and adolescence. Molecular Psychiatry.

7. Lee, J.J., Wedow, R., Okbay, A., Kong, E., Maghzian, O., Zacher, M., Nguyen-Viet, T.A., Bowers, P., Sidorenko, J., Linner, R.K., et al. (2018). Gene discovery and polygenic prediction from a genome-wide association study of educational attainment in 1.1 million individuals. Nat. Genet. 50, 1112–1121.

8. Luciano, M., Hagenaars, S.P., Davies, G., Hill, W.D., Clarke, T.-K., Shirali, M., Harris, S.E., Marioni, R.E., Liewald, D.C., Fawns-Ritchie, C., et al. (2018). Association analysis in over

329,000 individuals identifies 116 independent variants influencing neuroticism. Nat. Genet. 50, 6–11.

9. Demontis, D., Walters, R.K., Martin, J., Mattheisen, M., Als, T.D., Agerbo, E., Baldursson, G., Belliveau, R., Bybjerg-Grauholm, J., Bækvad-Hansen, M., et al. (2019). Discovery of the first genome-wide significant risk loci for attention deficit/hyperactivity disorder. Nat. Genet. 51, 63–75.

10. Pardiñas, A.F., Holmans, P., Pocklington, A.J., Escott-Price, V., Ripke, S., Carrera, N., Legge, S.E., Bishop, S., Cameron, D., Hamshere, M.L., et al. (2018). Common schizophrenia alleles are enriched in mutation-intolerant genes and in regions under strong background selection. Nat. Genet. 50, 381–389.
